# The evolution of ependymin-related proteins

**DOI:** 10.1186/s12862-018-1306-y

**Published:** 2018-12-04

**Authors:** Carmel McDougall, Michael J. Hammond, Simon C. Dailey, Ildiko M. L. Somorjai, Scott F. Cummins, Bernard M. Degnan

**Affiliations:** 1 Centre for Marine Science, School of Biological Sciences, The University of Queensland, Brisbane, Queensland 4072 Australia; 2 Australian Rivers Institute, Griffith University, Nathan, Queensland 4111 Australia; 3 GeneCology Research Centre, University of the Sunshine Coast, Maroochydore DC, Queensland 4558 Australia; 4 Gatty Marine Laboratory, Scottish Oceans Institute, University of St Andrews, St Andrews, KY16 8LB UK; 5 Biomedical Sciences Research Complex, University of St Andrews, North Haugh, St Andrews, KY16 9ST UK

**Keywords:** Ependymin, EPDRs, MERPs, Gene duplication, Gene loss

## Abstract

**Background:**

Ependymins were originally defined as fish-specific secreted glycoproteins involved in central nervous system plasticity and memory formation. Subsequent research revealed that these proteins represent a fish-specific lineage of a larger ependymin-related protein family (EPDRs). EPDRs have now been identified in a number of bilaterian animals and have been implicated in diverse non-neural functions. The recent discoveries of putative EPDRs in unicellular holozoans and an expanded EPDR family with potential roles in conspecific communication in crown-of-thorns starfish suggest that the distribution and diversity of EPDRs is significantly broader than currently understood.

**Results:**

We undertook a systematic survey to determine the distribution and evolution of EPDRs in eukaryotes. In addition to Bilateria, EPDR genes were identified in Cnidaria, Placozoa, Porifera, Choanoflagellatea, Filasterea, Apusozoa, Amoebozoa, Charophyta and Percolozoa, and tentatively in Cercozoa and the orphan group Malawimonadidae. EPDRs appear to be absent from prokaryotes and many eukaryote groups including ecdysozoans, fungi, stramenopiles, alveolates, haptistans and cryptistans. The EPDR family can be divided into two major clades and has undergone lineage-specific expansions in a number of metazoan lineages, including in poriferans, molluscs and cephalochordates. Variation in a core set of conserved residues in EPDRs reveals the presence of three distinct protein types; however, 3D modelling predicts overall protein structures to be similar.

**Conclusions:**

Our results reveal an early eukaryotic origin of the EPDR gene family and a dynamic pattern of gene duplication and gene loss in animals. This research provides a phylogenetic framework for the analysis of the functional evolution of this gene family.

**Electronic supplementary material:**

The online version of this article (10.1186/s12862-018-1306-y) contains supplementary material, which is available to authorized users.

## Background

Ependymins are secreted glycoproteins comprised of approximately 200 amino acids with four conserved cysteines. They were first discovered to be associated with the ependyma of the brain in fish where they appear to be required for the formation of long-term memory and neuronal regeneration [1–3]. Although long believed to be fish-specific, subsequent discoveries of ependymin-related proteins (EPDRs) in mammals [named ‘mammalian ependymin-related proteins’ (MERPS) [4] or in humans 'upregulated in colorectal cancer gene-1' (UCC1) [5]], amphibians [4] and echinoderms [6] demonstrated that the distribution of the gene family was much broader than originally thought.

These studies also revealed that EPDRs might perform roles outside the central nervous system. Although the specific functions of MERPs are unclear, they are highly expressed in brain, heart, skeletal muscle, kidney and hematopoetic cells [4, 7], and in some tumours [5]. UCC1 is involved in Dupuytren’s disease, a connective tissue disorder, in which it affects the collagen contractility of fibroblasts [8]. In sea cucumbers, EPDR genes are expressed in several tissues and are upregulated during regeneration of the eviscerated gut [6, 9]. The expression of these EPDR genes in non-brain tissues and the implication of their involvement in diverse processes indicated that the functional diversity of this protein family had been underestimated.

It was within this context that Suárez-Castillo and García-Arrarás conducted the first systematic analysis of EPDRs in animals, aided by the growing availability of expressed sequence tag (EST) projects [10]. Not only did they detect EPDRs in non-deuterostome bilaterians, they also identified additional piscine EPDRs that were expressed in non-brain tissues. Phylogenetic analyses of this newly-collated dataset of EPDR sequences revealed the presence of four major clades. The first group, the ‘Fish Brain’ group, contained the original, brain tissue-specific ependymin sequences. Sister to this group was a second fish-specific group, named ‘Fish Tj’, containing other fish EPDRs that were expressed in non-brain tissues. The third major group contained the previously identified ‘MERPS’, as well as echinoderm, amphibian, bird and fish representatives. Finally, the analysis revealed the likely presence of a fourth ‘basal’ group of EPDRs that contained sequences from molluscs, amphioxus, and ascidians. This analysis and the presence of fish sequences in three of the four clades led the authors to propose that gene duplication, likely from an ancestral ‘basal’ EPDR, followed by degeneration and complementation of gene sequences (the DDC model [11]) resulted in the presence of multiple, functionally-diverse EPDR genes in vertebrates. The placement of some sequences, however, was not entirely congruent with this scenario; namely, the position of echinoderm sequences within the MERP clade, and the grouping of oyster (bivalve mollusc) sequences with that of amphioxus rather than with the other molluscs, which, themselves, appeared as a sister group to the poorly supported ‘basal’ EPDRs.

Since the publication of this analysis, potential EPDRs have been reported in non-metazoan unicellular and multicellular unikont (Holozoa, Fungi, Apusozoa and Amoebozoa) relatives [12, 13], suggesting EPDRs are more ancient than previously thought. A large duplication of EPDR genes has also recently been reported in echinoderms [14]. The crown-of-thorns starfish has a large EPDR gene family expansion with at least some of the EPDR proteins being secreted outside the animal during aggregation or in response to the presence of a predator, suggesting a role in conspecific signalling [14]. Phylogenetic analysis of echinoderm EPDRs revealed that sea cucumbers and other echinoderms have multiple EPDR genes, suggesting they may perform additional functions in these species. Outside deuterostomes, a number of transcriptome and proteome-based studies, predominately in molluscs, have linked EPDRs with a variety of functions. ‘S*ometsuke’*, an EPDR described in the abalone (a gastropod mollusc), has a role in shell biomineralisation and pigmentation [15, 16], and EPDRs have also been found in mantle transcriptomes and shell proteomes in other mollusc species [17–19]. In addition, EPDRs have been implicated in gastropod larval development [20], are upregulated in response to toxins and metal pollution in bivalves [21–24], and are differentially regulated during pathogen challenge trials in bivalves [25–27] and in response to environmental stressors in both bivalves and gastropods [28–30]. Together, these studies are consistent with EPDRs having a diversity of functions, some of which appear to be lineage-specific.

To further understand the structural and functional evolution of the EPDR family we performed a systematic survey for EPDRs in eukaryotes and conducted phylogenetic analyses to investigate their relationships. We found further support for this gene family evolving before the origin of Metazoa. The EDPRs can be divided into two major clades that can be distinguished by different patterns of conserved residues, notably, different numbers of cysteine residues. This is consistent with these clades being functionally distinct. Mapping these EPDR clades onto an organismal phylogeny suggests these cysteine profiles can arise de novo within lineages, and that convergent evolution is possible. The absence of EPDR genes in many eukaryote clades demonstrates that the gene family has been lost multiple times in protist and metazoan lineages. In contrast, the gene family has undergone extensive independent duplication and divergence within several metazoan groups.

## Results

### Phylogenetic distribution of EPDRs

The ependymin gene family is represented in the Pfam database (PF00811) [31], where an estimation of its distribution is provided based upon searches of reference proteomes from the UniProt database [32]. These data suggest that EPDRs are present in metazoans and some unicellular eukaryotic proteomes. However, no EPDRs were reported from bacterial, viral or archaeal reference proteomes (numbering 82,518, 82,616, and 860 proteomes, respectively). We therefore restricted our analysis to eukaryotic taxa.

**Fig. 1:**
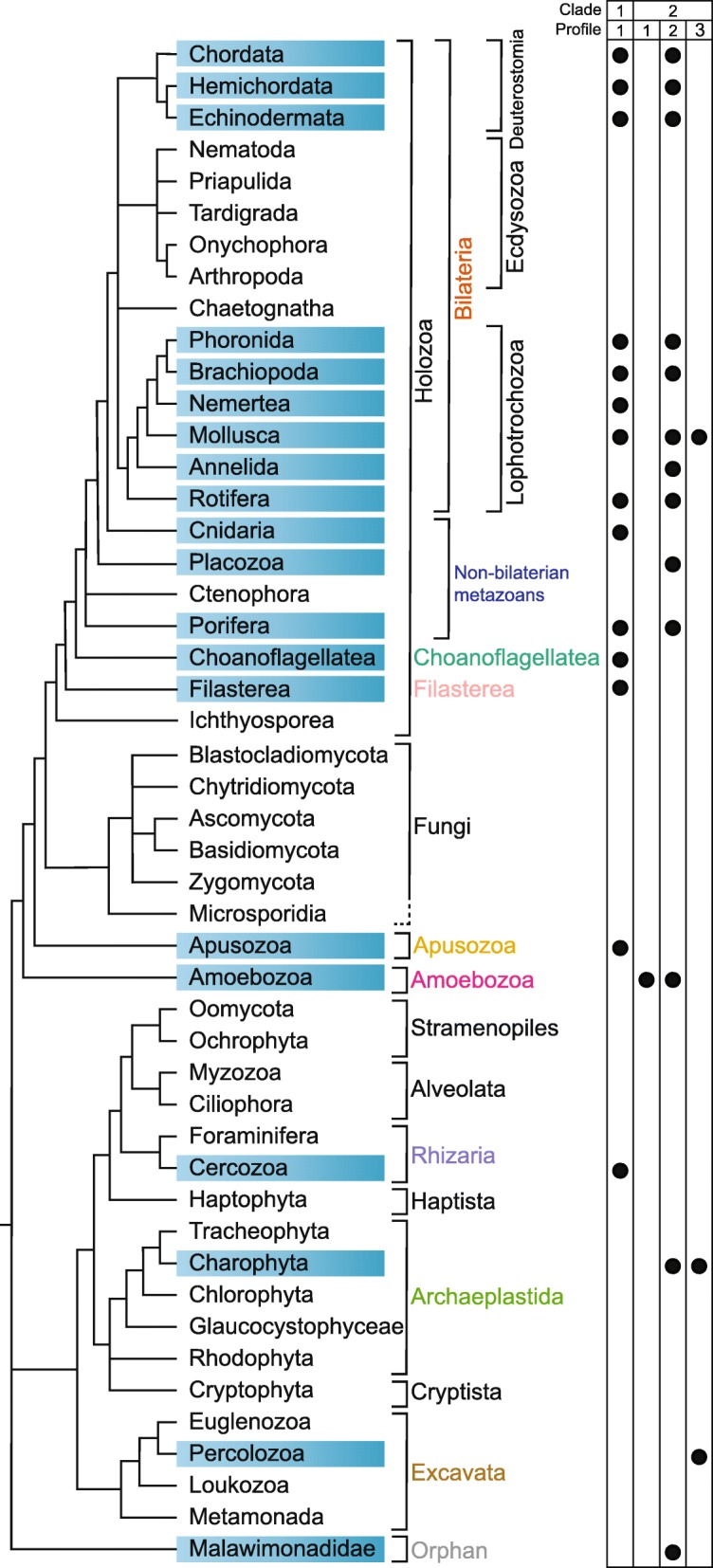
Distribution of EPDR proteins in Eukaryota. The cladogram to the left indicates the currently accepted phylogenetic relationships among taxa [38, 64–70]. The presence of EPDR sequences in particular groups is indicated by blue boxes. The clade membership (according to phylogenetic analysis) and profile membership (based upon conserved cysteine residue patterns) of each group is indicated in the schematic on the right

Systematic HMM searches of predicted proteins from eukaryotic genomes and transcriptomes identified 420 sequences and confirmed the existence of EPDRs outside Metazoa (Fig. 1, Additional file 1). Each of these sequences aligned to other EPDRs and contained characteristic highly conserved cysteine residues (see below); they were thus classified as true EPDRs (see Additional file 2 for a note on incorrectly predicted EPDR protein models in the genomes of several species). Specifically, EPDR genes were identified in both choanoflagellates and filastereans, in *Thecamonas trahens* (Apusozoa), in three dictyostelids and an acanthamoeban (Amoebozoa), in three charophytes (Archaeplastida), and in two percolozoans (Excavata). In each of these cases the presence of EPDR sequences is supported by both genome and transcriptome evidence and, in the case of *Dictyostelium discoideum*, by the presence of the EPDR gene on a scaffold containing other *D. discoideum* genes (i.e., it is unlikely to originate from contamination). In initial analyses, two potential EPDR sequences were identified by HMM searches in *D. discoideum*, both with e-values higher than the threshold used in this study. One of these aligned well with other EPDRs and was thus classified as a true EPDR. The second, which is identical to the sequence reported to be an ependymin by Pei and colleagues [13], did not contain the conserved pattern of EPDR cysteines and therefore was not classified as a member of this gene family.

Putative EPDR transcripts were also detected in the cercozoan *Paracercomonas marina* (Rhizaria) and *Malawimonas jakobiformis* (Malawimonadidae), although these designations must be treated with caution until the possibility of contamination can be discounted. The latter most probably accounts for the discovery of an EPDR in a root nodule transcriptome of the tracheophyte *Aeschynomene indica* given that this sample likely contained an assortment of other organisms, and that EPDR sequences were not found in other tracheophytes including those with whole genome data. This sequence was not investigated further in our analysis. It must be noted that sequence availability for many eukaryotic groups is poor, and sequenced organisms do not represent the entire diversity of these clades [33]. For this reason, it is likely that EPDRs will be detected from additional groups as more data become available. This caveat aside, no EPDRs were detected in a number of sequence-rich groups, including Fungi, Tracheophyta, Chlorophyta, Rhodophyta, Myzozoa, and Euglenozoa.

Our survey also greatly extended the known EPDR gene complement in metazoans. The number of EPDR genes detected in genomes or transcriptomes varies greatly between species, with large expansions (15 EPDR genes or more) detected in the cephalochordate *Branchiostoma belcheri*, the asteroid echinoderms *Patiria miniata* and *Acanthaster planci*, the gastropod molluscs *Aplysia californica, Lottia gigantea* and *Haliotis asinina*, the bivalve mollusc *Crassostrea gigas*, the brachiopod *Lingula anatina,* and the sponge *Amphimedon queenslandica* (Additional file 1). Other groups appear to have lost the gene family entirely. For example, no EPDRs could be found in ctenophores or in ecdysozoans.

**Fig. 2:**
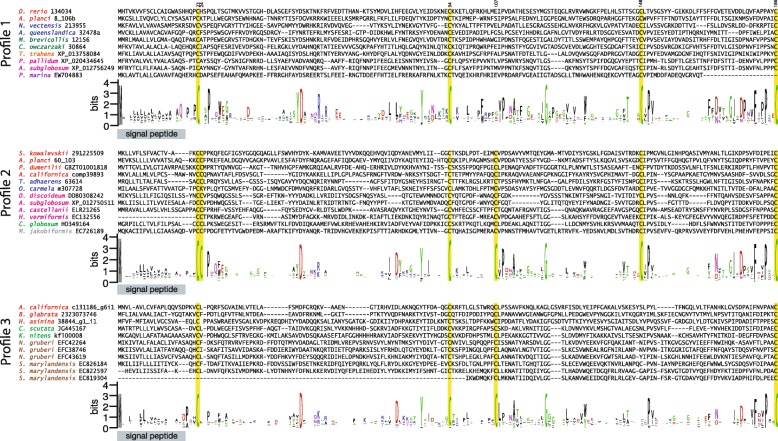
Alignment of representative EPDR protein sequences. Comparative alignments of representatives displaying each cysteine residue profile are shown*. D. rerio* 134034 is a fish ependymin and is included as a representative of profile 1. The signal peptide is indicated by a grey box, and conserved cysteine residues are highlighted in yellow and numbered at the top of the alignment. The sequence logos were calculated from the alignment of all detected EPDR proteins for each profile (see Additional file 4), and thus do not directly correspond to the representative sequences shown. The overall height of each column corresponds to the degree of conservation at that site, and the height of each letter corresponds to the overall frequency of that amino acid. The colouring of species names corresponds to the phylogenetic groups to which the species belong, and follows the scheme established in Fig. 1

Alignment of identified EPDR protein sequences revealed the presence of a conserved signal peptide, cysteine residues, and a number of other amino acids (Fig. 2, Additional files 3 and 4). Outside these residues very little sequence similarity can be observed in EPDRs from divergent taxa. The alignment also revealed the presence of three overall patterns of conserved cysteine residues within EPDR genes (profiles 1, 2 and 3; compare sequence logos in Fig. 2). Three cysteines are conserved in all three profiles at positions 23, 94 and 186 of the alignment. Profile 1 has one additional conserved cysteine at position 148, profile 2 has three additional conserved cysteines at positions 24, 107 and 148, and profile 3 has one additional conserved cysteine at position 107. Overall protein length appears to be highly conserved across all three profile groups, with a median length of 213 amino acids (Additional file 5).

**Fig. 3:**
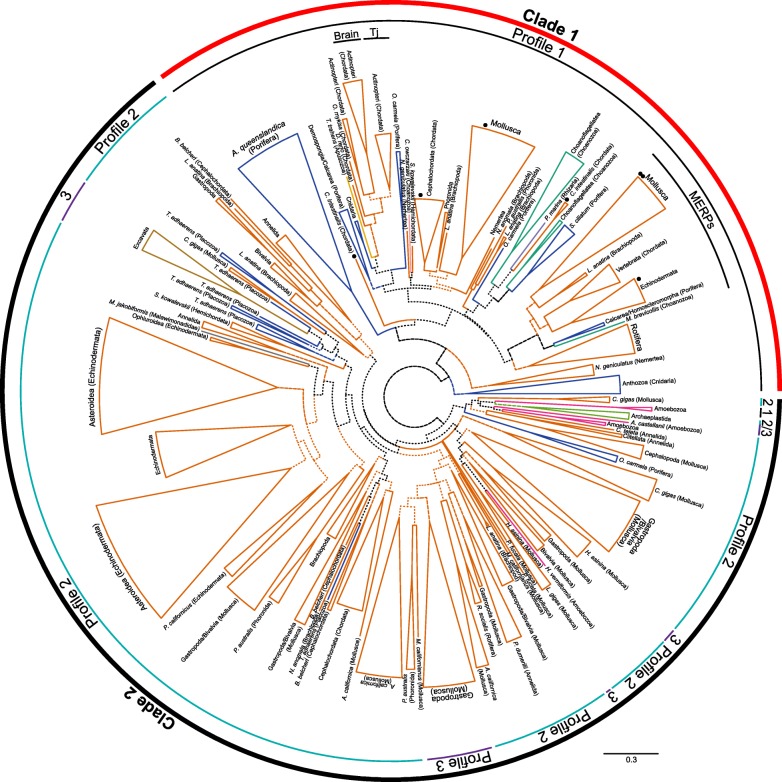
Summarised phylogenetic analysis of EPDR protein sequences. Colouring of the branches corresponds to the phylogenetic groups to which the species belong, and follows the scheme established in Fig. 1. Maximum likelihood tree, branches with high support from both analyses (maximum likelihood bootstrap values > 80 and Bayesian posterior probability values > 90) are indicated by a solid line, those with bootstrap values > 50 or posterior probability values > 70 are indicated by a dashed line, and those with lower support are indicated by a dotted line. The two major EPDR clades are indicated by the outer circle, the cysteine profile displayed by the sequences is indicated by the inner circle. Major clades identified by Suárez-Castillo and García-Arrarás [10] are also indicated (Brain, Tj, and MERPs), including those proposed to belong to the ‘basal’ clade (black dots). The scale bar indicates the number of substitutions per site. For a complete tree including support values and individual sequence names refer to Additional file 6

Phylogenetic analyses (maximum likelihood and Bayesian) generally produced good support for taxon-specific gene clusters, but poor support for early diverging branches (Fig. 3, Additional file 6). Bayesian analysis did not reach convergence after 30 million generations, and therefore the results must be treated with caution. The poor support is not unexpected given the low degree of overall sequence conservation among EPDR sequences [10]. The analyses did separate EPDR protein sequences into two major clades, with some support (maximum likelihood ML bootstrap value 14, Bayesian posterior probability 74%). However, this support value increased in phylogenetic analyses with a reduced dataset in which potentially problematic taxa (e.g., protists, placozoans and brachiopods) were removed (Additional file 7, maximum likelihood ML bootstrap value 65, Bayesian posterior probability 96%, with convergence reached in the Bayesian analysis). The division of EPDR sequences into these two major clades is also supported by the patterns of cysteine residues, with members of one clade (containing the originally described piscine ‘brain’ ependymins and ‘Tj’ EPDRs, the MERPS, and EPDRs from protostomes, Choanoflagellatea, Filasterea, Apusozoa, and *P. marina* (Rhizaria)) displaying profile 1, and members of the second clade (containing metazoan, amoebozoan, charophyte, percolozoan and malawimonad sequences) displaying all three profiles. We refer to these clades as ‘clade 1’ and ‘clade 2’, respectively (Fig. 3). Some sequences deviate from these overall patterns, having lost one or more cysteine residues, or gained additional ones (e.g., two *Capitella teleta* sequences are lacking two cysteines, and the MERPs possess an extra cysteine, Additional files 3 and 4), although their clade membership is evident both from the alignment and the phylogenetic analysis. It is therefore likely that these clades reflect a deep branching of EPDRs into two distinct lineages. Determining the point in evolutionary history at which these clades split is problematic, due to the placement of the *P. marina* (Rhizaria) sequence within clade 1 that otherwise consists solely of EPDRs from unikont taxa. Confirmation of the existence of EPDRs in Rhizaria via the sequencing of EPDR sequences in additional species and/or genomes will clarify this issue.

Determining the evolutionary history of EPDRs within clade 2, which exhibit all three cysteine patterns, is similarly problematic. Within this clade the type 1 profile is restricted to two amoebozoan sequences (the remaining amoebozoan sequences are type 2, Fig. 3 and Additional file 6). Likewise, the type 3 profile is not common, being restricted to charophyte (Archaeplastida) and excavate taxa, as well as to a small subset of molluscan sequences. Given that these molluscan sequences are the only example of the type 3 profile in unikonts, and that the clades containing them are nested within type 2 containing clades (mostly with good support, Fig. 3 and Additional file 6), it is likely that this pattern arose convergently in molluscs from a type 2 ancestral gene. A similar situation exists in the charophyte taxa, where all three EPDR sequences form a monophyletic group (in maximum likelihood but not Bayesian analyses), but two of the sequences are type 3 and the third is type 2 (Fig. 3). Unless this discrepancy is due to contamination, this may represent an additional case of convergent evolution.

### EPDR duplications in metazoan lineages

The phylogenetic analysis of EPDR protein sequences revealed independent large gene family expansions in a number of metazoan taxa, as outlined above. Seventeen EPDR genes were identified from the demosponge *Amphimedon queenslandica* genome and formed a well-supported group within clade 1 in the phylogenetic analysis (Fig. 3, Additional file 6 clade A). Only two EPDRs were identified from ESTs of a second demosponge, *Suberites domuncula.* These sequences were also placed within clade 1, but clustered with two of the 8 EPDRs from the calcareous sponge *Sycon ciliatum* (Additional file 6 clade B)*.* A fourth sponge species, the homoscleromorph *Oscarella carmela,* has both clade 1 and clade 2 EPDR representatives (Additional file 6 clades C, D). One of the clade 1 sequences clusters with a *S. ciliatum* sequence within the MERPs, while the positions of the remaining clade 1 sequences are poorly resolved. These results indicate that at least one ancestral clade 1 gene was present in the sponge ancestor, and strongly suggest that the *A. queenslandica* genes are the result of taxon-specific duplications, possibly functioning in a different manner to those in other sponge classes.

Numerous EPDR duplications have also occurred in lophotrochozoan lineages. Molluscan EPDR representatives form numerous clades throughout the tree, in both clade 1 and clade 2. Some groups contain representatives from most of the surveyed gastropod and bivalve taxa, indicating that some diversification occurred prior to their divergence (e.g., clade E, Additional file 6). Others are restricted to bivalves, gastropods, or to individual species (e.g., clade F, Additional file 6), indicating post-divergence diversification. All cephalopod sequences fall into a single group within clade 2 and do not cluster with other molluscan sequences, reflecting different patterns of evolution within this class (Additional file 6 clade G). Another large expansion has occurred within the brachiopod *Lingula anatina*, and representative EPDRs from this species are likewise distributed broadly throughout the tree, generally as well-supported clusters of 2–5 sequences. Both molluscs and brachiopods have representative sequences placed within the extended MERPs, elsewhere in clade 1, and in various (poorly-supported) groups within clade 2, indicating the presence of at least two clade 1 genes and one (probably more) clade 2 gene in the last common ancestor of lophotrochozoans.

A number of expansions have also occurred within deuterostome lineages. As observed previously [14], the EPDR repertoire of Echinodermata is the result of multiple duplication events throughout the evolution of this lineage, featuring large duplications of clade 2 EPDRs, particularly within asteroid lineages (e.g., clade H, Additional file 6). Within cephalochordates, two separate gene expansions appear to have occurred within the *Branchiostoma* lineage. With the exception of one divergent sequence, the EPDR genes are split between two major clades, one that falls within clade 1 (Additional file 6 clade I), and the other within clade 2 (Additional file 6 clade J, not monophyletic). A number of close 1:1 orthologues can be observed between the species; therefore these expansions took place, at least in part, prior to speciation. No MERP candidate can be found within the two *Branchiostoma* species; given the presence of echinoderm and vertebrate sequences within this group, we can infer that this is due to loss. Based on the distribution of deuterostome EPDRs, it can be inferred that the last common ancestor possessed one MERP, a second clade 1 EPDR, and at least one clade 2 EPDR gene.

Duplicated genes are found in clusters within the genomes of a number of the investigated species, including *A. queenslandica*, *B. belcheri*, and *A. planci* (Additional file 8), and clustered genes tended to group together in the phylogenetic analysis (e.g., *A. planci* genes within clade K, Additional file 6), suggesting that expansion of this gene family has occurred via tandem duplication in several lineages. Intra-class synteny analysis was performed between species with whole genome information where possible to determine whether EPDR genes are conserved within larger gene clusters, and whether this information could be used to aid in evolutionary reconstructions. No synteny was observed among any of the species compared (data not shown).

### Sequence characteristics of EPDR proteins

**Fig. 4:**
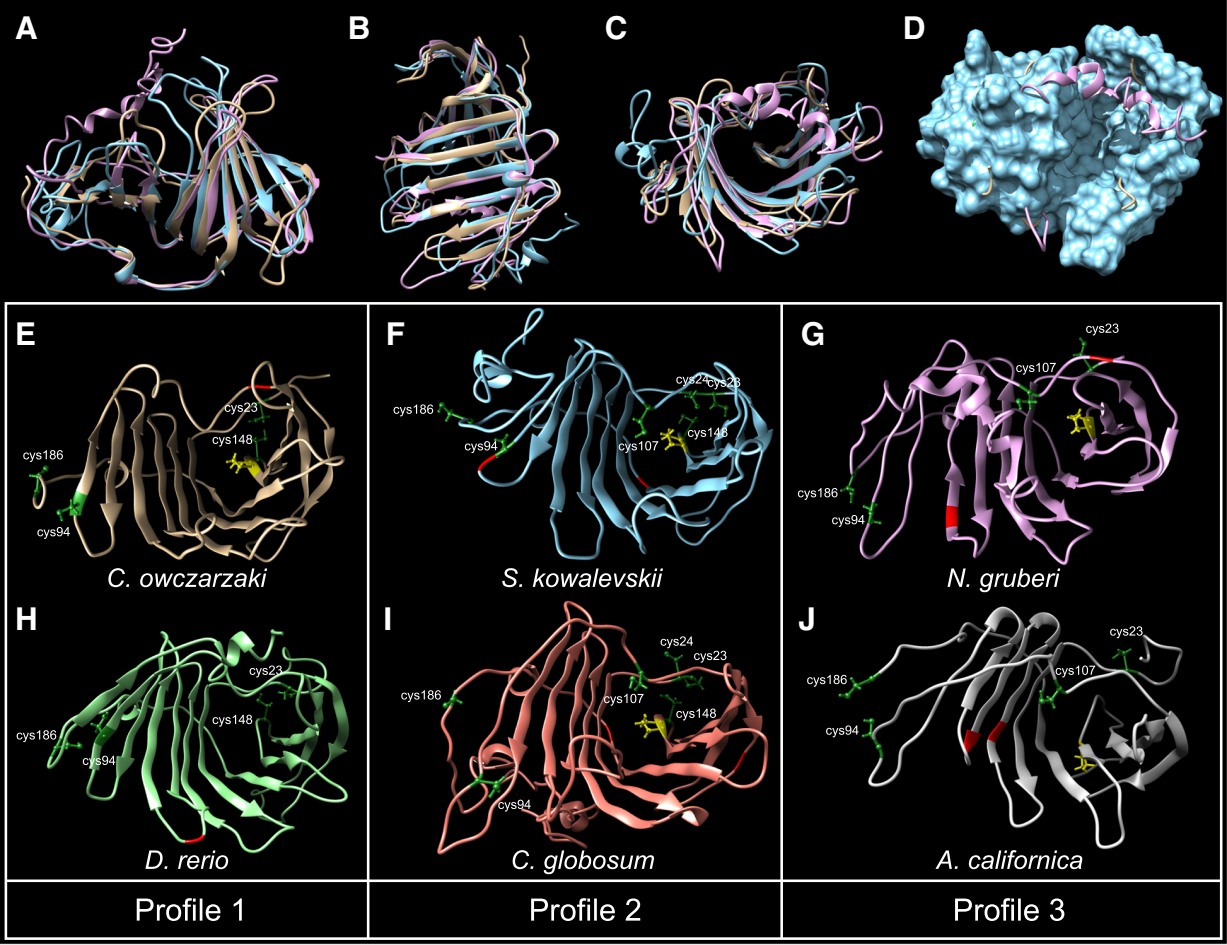
Predicted 3D structure of representative EPDRs. **a-c**; three different views of *Capsaspora owczarzaki* 30864 (clade 1, profile 1, gold), *Saccoglossus kowalevskii* 291225509 (clade 2, profile 2, blue) and *Naegleria gruberi* EFC42264 (clade 2, profile 3, pink) EPDR sequences, superimposed. All three sequences are predicted to form a twisted beta-sheet structure that surrounds a central pocket (visible in C). **d**. Surface rendering of C, based on the *S. kowalevskii* sequence. The shading in the centre indicates the location of the pocket. Panels E-J present individual predicted 3D structures of *C. owczarzaki* 30864 (**e**), *S. kowalevskii* 291225509 (**f**), *N. gruberi* EFC42264 (**g**), *Danio rerio* 134034 (a fish brain ependymin; **h**), *Chaetosphaeridium globosum* HO349164 (**i**), and *Aplysia californica* (**j**), respectively, in a similar orientation. Highly conserved cysteine residues are indicated in green. The highly conserved proline (position 150 of alignment) is located at the bottom of the pocket and is indicated in yellow, except for *D. rerio* 134034 in which it is absent. Predicted glycosylation sites are indicated in red. All sequences display similar predicted structures, however there appears to be some divergence in the portion of the protein distal to the pocket (left of figure), particularly in profile 3 proteins

The conservation of EPDR protein hydropathy profiles observed in previous studies has led authors to suggest that the overall functionality of the protein may be maintained despite sequence divergence [4, 34]. To investigate this further, we undertook structural predictions of representative EPDRs using I-TASSER [35]. I-TASSER compares query sequences to known structural templates and generates a topology by combining likely structures from aligned regions with predicted folding for unaligned regions. The program also identifies potential ligand-protein interactions. Although the overall confidence scores of the predicted models were low (Additional file 9), alignment in UCSF Chimera [36] revealed a high degree of similarity in predicted structures of proteins from both clades and with all three conserved cysteine profiles (Fig. 4a-d). These sequences are predicted to form a folded beta-sheet structure and two linker regions, creating a deep pocket within the centre of the protein (Fig. 4c-d). Ligand-protein prediction reveals the potential for ligand docking within this site, and a number of potential ligands were predicted, albeit with low confidence (Additional file 9).

Mapping the conserved cysteine residues on to each predicted structure indicates that some are likely to participate in cysteine bonds. Although I-TASSER does not explicitly model disulphide bridges, close localisation of cysteine residues may indicate that bonds occur. In cysteine profile 1, disulphide bridges may form between cysteines 94 and 186, and also between cysteines 23 and 148 (Fig. 4e, h). The former bond would anchor the C-terminal tail of the protein, whereas the latter would occur at the bottom of the protein pocket. Cysteine profile 2 contains two additional cysteines at position 24 (also at the bottom of the pocket), and at position 107, which appears to be within the pocket itself (Fig. 4f, i). There is a larger distance between these additional cysteines, therefore they may not be involved in cysteine bond formation. Profile 3 is similar to profile 1, except that it has a cysteine at position 107 (within the pocket) rather than at position 148 (at the bottom of the pocket) (Fig. 4g, j). It is unlikely that these two residues would form a bond, based on their predicted localisations. Other, non-cysteine residues that are highly conserved within EPDRs may also occupy important locations within the protein. One example is the highly conserved proline residue at position 150; this proline is situated in the loop that forms the floor of the pocket, and may influence ligand binding (yellow residue in Fig. 4e-j), although this residue is not absolutely conserved (i.e. there is no conserved proline in this position in *D. rerio* 134034, Fig. 4h; despite other ‘Fish Brain’ ependymins possessing the conserved residue).

Previous authors have reported the presence of conserved glycosylation sites 3 and 27 amino acids upstream of the second conserved cysteine in fish ependymin proteins, and many of their functional properties have been attributed to their N-linked carbohydrates [2, 10, 34, 37]. Putative glycosylation sites have also been identified in mammalian and echinoderm EPDRs; however, the positions of these sites are not conserved with those of piscine ependymins [6, 7]. We found predicted N-glycosylation sites in all but 30 of the EPDR sequences surveyed (Additional file 6, sequences indicated by a star); five of these were from ESTs that appeared incomplete. All taxa possessing EPDR members that do not have predicted N-glycosylation sites have additional EPDR genes that do, except for *D. discoideum*. Although the number and position of predicted glycosylation sites is not conserved (red regions in Fig. 4e-j), their presence in the majority of EPDR proteins investigated suggests that N-linked carbohydrates are functionally important.

## Discussion

The discovery of EPDRs in non-metazoan eukaryote taxa reveals that the gene family arose much earlier than previously thought. Given that relationships among major eukaryotic groups are still uncertain [33, 38], it is difficult to reconstruct the earliest evolutionary history of EPDR genes, i.e., whether the patchy distribution of EPDRs in these groups is the result of gene loss or the evolution of the gene family after the divergence of the major clades. In addition, the presence of single EPDR sequences in transcriptomes from single rhizarian and malawimonad species is problematic; it is unclear if they are contamination. This distinction is critical for reconstruction of the evolutionary history of this gene family, especially given that they possess sequence characters otherwise found only in unikont taxa. Horizontal gene transfer could have occurred, and careful analysis of whole genome data from these protist lineages will be required when they become available.

The phylogenetic analysis presented here, although weakly supported, is congruent with that conducted by Suárez-Castillo and García-Arrarás [10]. Our larger dataset, facilitated by increased availability of sequences, reveals that the discrepancies detected in this earlier study (particularly within the ‘basal’ group) are the result of incomplete sampling. Clade 2 sequences were not included by Suárez-Castillo and García-Arrarás in their analysis. This may reflect the absence of these sequences in the NCBI database at the time and/or differences in search strategy (i.e., HMM searches as performed here, in contrast to BLAST performed in the previous study). We argue that the overall similarity in sequence length, the presence of shared highly conserved residues in comparable positions (including the three ultra-conserved cysteines), and the similar predicted 3D structures suggest that clade 1 and clade 2 EPDRs arose from a single ancestral gene.

**Fig. 5:**
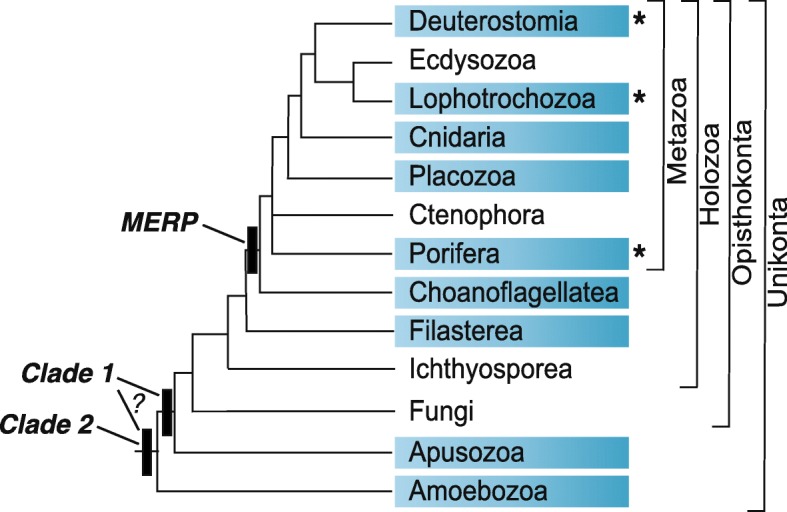
Proposed evolution of the EPDR family. The presence of EPDR sequences in particular groups is indicated by blue boxes, and the evolutionary origin of major EPDR clades is depicted by solid bars. The unikont ancestor likely possessed both clade 1 and clade 2 EPDRs, however it is possible that clade 1 EPDRs arose later, in the opisthokont ancestor. Major expansion of EPDR genes occurred in lineages indicated by an asterisk

Our analysis allows us to draw some conclusions about the ancestral EPDR complement in unikonts (Fig. 5). The existence of a MERP representative in a broad range of holozoans, including choanoflagellates, sponges, echinoderms, molluscs, brachiopods and chordates suggests that their common ancestor possessed a MERP gene. MERPs often exist as a single copy within genomes, and MERPs are the only EPDR present in vertebrates. Despite this, duplications have occurred in some lineages, including brachiopods, sea urchins and some gastropods (Fig. 3). No MERP was found in the apusozoan species investigated here or in other eukaryotes, suggesting that this EPDR clade arose in the holozoan ancestor. In addition to MERP genes, many of these groups also possess additional clade 1 EPDRs, indicating that the ancestral holozoan likely possessed at least two clade 1 EPDRs. The presence of a clade 1 gene in the apusozoan *Thecamonas trahens* suggests that this gene clade may have been present in the unikont ancestor; whether or not it is more ancient will rest upon whether the rhizarian sequence is contamination, or the result of convergent evolution or lateral gene transfer. The unikont ancestor also possessed a clade 2 EPDR, which has undergone a large number of duplications in some metazoan lineages. Given the presence of clade 2 EPDRs in both unikonts and a number of protist groups, it is likely that this clade represents the ancestral EPDR type.

Our analysis also reveals the dynamic nature of evolution of EPDRs in metazoans. Independent gene duplication events have occurred in numerous lineages, including sponges, molluscs, echinoderms and cephalochordates, and multiple tandem gene duplication events appear to have occurred at different points during molluscan and echinoderm evolution.

The underlying reason for these duplication events, and for the retention of multiple EPDR genes, is unknown. It is possible that these gene duplicates have undergone divergence and functional complementation (DDC), as has been suggested for vertebrate EPDRs [10], although confirmation awaits better understanding of their functions. Equally curious is the apparent loss of the EPDR gene family entirely from ecdysozoans and ctenophores. These losses indicate that EPDR genes are not performing functions that are essential for the survival of eukaryotic organisms, or that these functions can be compensated for by other mechanisms.

Based on prior reports in the literature, the range of functions performed by EPDRs is extraordinarily diverse. This may be attributable to particular biochemical or structural properties of EPDRs that are applicable in many different functional contexts. For example, the involvement of these proteins in fish memory consolidation [1], fish optic nerve regeneration [39], sea cucumber intestinal regeneration [6], and human fibroblast contractility [8] may be due to the interaction of EPDRs with components of the extracellular matrix (including collagen [40]) in a calcium-dependent manner [41]. Similar properties may underlie the co-option of EPDRs into the molluscan shell [15] (McDougall and Degnan, unpublished). Additionally, structural features of EPDRs can activate signalling pathways, as peptide fragments with different amino acid sequences that originate from the same region of fish and human EPDRs can activate AP1 and the JNK signalling pathway in cell culture via an unknown mechanism [42, 43]. A signalling role is also likely for the crown-of-thorns starfish *A. planci* EPDRs that are released into the water column by aggregating or alarmed animals [14]. Therefore, EPDRs may act as ligands themselves, in addition to being able to bind other ligands within their central pocket. Additional biochemical properties found for fish ependymins are also likely to be important for EPDR function, including the ability to form disulphide-bound dimers [44] and higher molecular weight aggregates [45, 46], the presence of N-linked carbohydrates that confer Ca^2^+ or cell-cell/cell-matrix binding ability [2, 3], and the ability to change structural conformation in the presence of Ca^2^+ [47]. Discovering whether these properties are associated with the conserved cysteine residues characteristic of the EPDRs, and how they relate to each of the three conserved profiles identified here, will be an interesting avenue of future research.

## Conclusions

The analyses presented here have revealed that the EPDR gene family arose early in eukaryotic evolution and is substantially larger than previously thought. We also identify a previously unrecognised clade of EPDR sequences with distinct patterns of conserved cysteine residues. Despite significant primary sequence divergence outside the core conserved residues, predicted 3D structures of EPDRs are similar, even among the three profile types. EPDRs are evolutionarily dynamic, having undergone independent expansions in multiple animal lineages, while being entirely lost from others. Although widely distributed, the structure and function of these proteins are largely unknown. Understanding the structure-function relationship of EPDRs will likely provide an explanation for the apparent proclivity of this family to be co-opted into diverse and lineage-specific roles in the extracellular environment, ranging from memory formation to biomineralization to conspecific communication.

## Methods

### Identification, phylogenetic analysis and characterisation of EPDR genes

Predicted protein datasets were downloaded from a range of species for which whole genome data are available. This dataset was supplemented by transcriptomes of key taxa; sequence reads were downloaded from NCBI nucleotide, protein, TSA or SRA databases, assembled if necessary using Trinity vr20131110 [48], and translated where required using TransDecoder (within the Trinity suite) with a minimum peptide length of 50 amino acids. Details of all data sources are provided in Additional file 10. These datasets were then searched for the ependymin domain using HMMER3.1 [49] and the ependymin pHMM (Pfam [31] accession PF00811.14) with an e-value cutoff of 1e-05. For each species, all identified sequences (Additional file 11) were aligned using COBALT, a constraint-based tool that performs alignments aided by information about protein domains [50], with a gap penalty of 13 (all other settings were default). Alignments were viewed and manually adjusted within AliView [51]. Identical or very similar sequences were removed, as were incomplete sequences that were missing a large portion of the conserved EPDR domain (Additional file 3). The alignment was then trimmed to remove apparent sequence-specific insertions and the divergent 3′ portion of the sequences (Additional file 4).

Maximum likelihood trees were produced using RAxML 7.7.6 [52], with automatic model selection (PMB model selected) and 100 replicates of nonparametric bootstrapping. Bayesian analysis was performed using MrBayes 3.2.6 [53, 54], using automatic (mixed) model selection (Blosum) with sampling every 10,000 generations. The analysis was run for 30 million generations and did not reach convergence (standard deviation of split frequencies 0.121180). Topologies of the resulting phylogenetic trees were largely congruent.

Sequences were analysed using SignalP 4.0 [55] to predict signal sequences, and NetNGlyc 1.0 [56] to predict glycosylation sites. Sequence logos were created for each class of EPDR using WebLogo [57]. Predictions of 3D structure and potential ligand binding sites were performed using I-TASSER [35], and resulting PDB model viewing and alignment was performed in UCSF Chimera [36]. Violin plots of protein sequence lengths were produced in R [58] using the program ggplot2 [59].

Synteny analysis was performed either using JGI’s inbuilt synteny analysis tool (for JGI hosted genomes) [60], or by reciprocal BLAST of neighbouring gene models using the BLAST function provided by species-specific genome browsers.

### Identification and characterisation of *Branchiostoma belcheri* ependymin-related gene sequences

Initial BLASTP searches of the *B. belcheri ‘*v18h27.r3’ reference predicted protein set [61] with EPDR proteins identified several EPDR-like protein models. However, when the genomic loci associated with these were searched using TBLASTN, it was evident that some exons had been omitted or misattributed. In one instance, two distinct EPDR-like loci were conflated into a single protein model, and conversely, some models were partial sequences originating from a single locus. Similar issues were previously encountered with *A. planci* EPDR gene models [14]. The proteins used here therefore are built from manually identified exons, but take their name from the original model(s). The EST database associated with this genome project (‘B.bel_xiamen_beihai.merged454EST’) provided at least partial and in most instances full-length support for these curated EPDR proteins. The manually curated protein sequences are available in Additional file 11. For a note on incorrectly predicted EPDR sequences see Additional file 2.

### Identification and characterisation of *Amphimedon queenslandica* ependymin-related gene sequences

As for *B. belcheri,* several *A. queenslandica* EPDR gene models appeared to be incorrect based upon the presence of multiple ependymin domains within a model, the poor alignment of these sequences against other EPDRs, and/or on the poor alignment of sequenced transcripts to the models when viewed on the genome browser [62, 63]. Where possible, transcript information was used to identify the correct intron/exon architecture of the genes in the genome assembly; where no transcripts were present, translations of all three frames were investigated for alternative architectures and selected if they possessed canonical splice donor/acceptor sites and their use resulted in a better alignment. The manually curated protein sequences are available in Additional file 11.

### Identification and characterisation of *Haliotis asinina* ependymin-related gene sequences

*H. asinina* EPDR sequences were identified by HMM searches on predicted open reading frames from an in-house Trinity assembly of reads from pooled mantle tissue of six juvenile abalone. The library was sequenced on 1/6th of a lane on an Illumina HiSeq 2000 (2 X 100 bp reads). Raw reads have been deposited into the NCBI Sequence Read Archive, BioProject ID PRJNA386701. For this transcriptome, assembly was performed without normalization. Assembled *H. asinina* EPDR transcripts are available in Additional file 11.

## Additional files

Additional file 1: Distribution of EPDR genes in Eukaryota. Results of EPDR survey by species, including total number of sequences searched, number of EPDRs found, and clade membership of detected EPDRs. (XLSX 59 kb)

Additional file 2: Note on incorrectly predicted gene models. Description of Data: Description of incorrectly predicted gene models within several metazoan genomes. (PDF 684 kb)

Additional file 3: Full ependymin alignment, untrimmed. Description of Data: Alignment of all 420 sequences analysed in this study, untrimmed. (FA 287 kb)

Additional file 4: Full ependymin alignment, trimmed. Description of Data: Alignment of all 420 sequences analysed in this study, trimmed. The phylogenetic analysis was performed using this alignment. (FA 84 kb)

Additional file 5: Distribution of EPDR protein length. Description of Data: Violin plot displaying the range of sequence lengths exhibited by EPDR proteins. (PDF 837 kb)

Additional file 6: Results of phylogenetic analysis. Description of Data: Cladogram of the phylogenetic tree presented in Fig. 3, including bootstrap and posterior probability values. (PDF 1583 kb)

Additional file 7: Results of phylogenetic analysis on reduced dataset. Description of Data: Summary tree of phylogenetic analysis conducted after the removal of problematic taxa. (PDF 424 kb)

Additional file 8: Clustering of EPDR genes in genomes. Description of Data: Schematic indicating clustering of EPDR genes on scaffolds in the *Acanthaster planci, Amphimedon queenslandica* and *Branchiostoma belcheri* genomes. (PDF 912 kb)

Additional file 9: Predicted model scores and ligands for representative EPDR sequences. Description of Data: Table showing clade membership, model C-Scores, and predicted ligands of selected EPDR sequences. (PDF 60 kb)

Additional file 10: Sources of sequence data used in this study. Description of Data: Excel file providing details of data sources, including web link and references, if applicable. (XLSX 65 kb)

Additional file 11: Sequences of all EPDR proteins analysed in this study. Description of Data: Fasta file containing protein sequences of all EPDRs used in this study, including manually curated *Amphimedon queenslandica* and *Branchiostoma belcheri* sequences. (FA 94 kb)

## Abbreviations

EPDR: ependymin-related protein
EST: Expressed sequence tags
HMM: hidden markov model
MERP: mammalian ependymin-related protein

## References

[1] Shashoua VE (1985). The role of brain extracellular proteins in neuroplasticity and learning. Cell Mol Neurobiol.

[2] Ganss B, Hoffmann W (1993). Calcium binding to sialic acids and its effect on the conformation of ependymins. Eur J Biochem.

[3] Pradel G, Schachner M, Schmidt R (1999). Inhibition of memory consolidation by antibodies against cell adhesion molecules after active avoidance conditioning in zebrafish. J Neurobiol.

[4] Apostolopoulos J, Sparrow RL, McLeod JL, Collier FM, Darcy PK, Slater HR, Ngu C, Gregorio-King CC, Kirkland MA (2001). Identification and characterization of a novel family of mammalian ependymin-related proteins (MERPs) in hematopoietic, nonhematopoietic, and malignant tissues. DNA Cell Biol.

[5] Nimmrich I, Erdmann S, Melchers U, Chtarbova S, Finke U, Hentsch S, Hoffmann I, Oertel M, Hoffmann W, Müller O (2001). The novel ependymin related gene UCC1 is highly expressed in colorectal tumor cells. Cancer Lett.

[6] Suárez-Castillo EC, Medina-Ortíz WE, Roig-López JL, García-Arrarás JE (2004). Ependymin, a gene involved in regeneration and neuroplasticity in vertebrates, is overexpressed during regeneration in the echinoderm *Holothuria glaberrima*. Gene.

[7] Gregorio-King CC, McLeod JL, Collier FM, Collier GR, Bolton KA, Van Der Meer GJ, Apostolopoulos J, Kirkland MA (2002). MERP1: a mammalian ependymin-related protein gene differentially expressed in hematopoietic cells. Gene.

[8] Staats KA, Wu T, Gan BS, O'Gorman DB, Ophoff RA (2016). Dupuytren's disease susceptibility gene, EPDR1, is involved in myofibroblast contractility. J Dermatol Sci.

[9] Zheng F-X, Sun X-Q, Fang B-H, Hong X-G, Zhang J-X (2006). Comparative analysis of genes expressed in regenerating intestine and non-eviscerated intestine of *Apostichopus japonicus* Selenka (Aspidochirotida: Stichopodidae) and cloning of ependymin gene. Hydrobiologia.

[10] Suárez-Castillo EC, García-Arrarás JE (2007). Molecular evolution of the ependymin protein family: a necessary update. BMC Evol Biol.

[11] Force A, Lynch M, Pickett FB, Amores A, Yan YL, Postlethwait J (1999). Preservation of duplicate genes by complementary, degenerative mutations. Genetics.

[12] King N, Westbrook MJ, Young SL, Kuo A, Abedin M, Chapman J, Fairclough S, Hellsten U, Isogai Y, Letunic I (2008). The genome of the choanoflagellate *Monosiga brevicollis* and the origin of metazoans. Nature.

[13] Pei J, Grishin NV (2012). Cysteine-rich domains related to frizzled receptors and hedgehog-interacting proteins. Protein Sci.

[14] Hall MR, Kocot KM, Baughman KW, Fernandez-Valverde SL, Gauthier MEA, Hatleberg WL, Krishnan A, McDougall C, Motti CA, Shoguchi E (2017). The crown-of-thorns starfish genome as a guide for biocontrol of this coral reef pest. Nature.

[15] Jackson DJ, McDougall C, Green K, Simpson F, Wörheide G, Degnan BM (2006). A rapidly evolving secretome builds and patterns a sea shell. BMC Biol.

[16] Marie B, Marie A, Jackson DJ, Dubost L, Degnan BM, Milet C, Marin F (2010). Proteomic analysis of the organic matrix of the abalone *Haliotis asinina* calcified shell. Proteome Sci.

[17] Mann K, Edsinger-Gonzales E, Mann M (2012). In-depth proteomic analysis of a mollusc shell: acid-soluble and acid-insoluble matrix of the limpet *Lottia gigantea*. Proteome Sci.

[18] Yarra T, Gharbi K, Blaxter M, Peck LS, Clark MS (2016). Characterization of the mantle transcriptome in bivalves: *Pecten maximus*, *Mytilus edulis* and *Crassostrea gigas*. Mar Genomics.

[19] Li H, Liu B, Huang G, Fan S, Zhang B, Su J, Yu D (2017). Characterization of transcriptome and identification of biomineralization genes in winged pearl oyster (*Pteria penguin*) mantle tissue. Comp Biochem Physiol Part D Genomics Proteomics.

[20] Heyland A, Vue Z, Voolstra CR, Medina M, Moroz LL (2010). Developmental transcriptome of *Aplysia californica*. J Exp Biol.

[21] Detree C, Núñez-Acuña G, Roberts S, Gallardo-Escárate C (2016). Uncovering the complex transcriptome response of *Mytilus chilensis* against saxitoxin: implications of harmful algal blooms on mussel populations. PLoS One.

[22] Song Q, Zhou H, Han Q, Diao X (2017). Toxic responses of *Perna viridis* hepatopancreas exposed to DDT, benzo(a)pyrene and their mixture uncovered by iTRAQ-based proteomics and NMR-based metabolomics. Aquat Toxicol.

[23] Chen H, Song Q, Diao X, Zhou H (2016). Proteomic and metabolomic analysis on the toxicological effects of benzo[a]pyrene in pearl oyster *Pinctada martensii*. Aquat Toxicol.

[24] Xu L, Ji C, Wu H, Tan Q, Wang W-X (2016). A comparative proteomic study on the effects of metal pollution in oysters *Crassostrea hongkongensis*. Mar Pollut Bull.

[25] Jiang F, Yue X, Wang H, Liu B (2017). Transcriptome profiles of the clam *Meretrix petechialis* hepatopancreas in response to Vibrio infection. Fish Shellfish Immunol.

[26] Allam B, Pales Espinosa E, Tanguy A, Jeffroy F, Le Bris C, Paillard C (2014). Transcriptional changes in Manila clam (*Ruditapes philippinarum*) in response to Brown ring disease. Fish Shellfish Immunol.

[27] Romero A, Forn-Cuní G, Moreira R, Milan M, Bargelloni L, Figueras A, Novoa B (2015). An immune-enriched oligo-microarray analysis of gene expression in Manila clam (*Venerupis philippinarum*) haemocytes after a *Perkinsus olseni* challenge. Fish Shellfish Immunol.

[28] Zhang Y, Sun J, Mu H, Li J, Zhang Y, Xu F, Xiang Z, Qian P-Y, Qiu J-W, Yu Z (2015). Proteomic basis of stress responses in the gills of the Pacific oyster *Crassostrea gigas*. J Proteome Res.

[29] Muraeva OA, Maltseva AL, Mikhailova NA, Granovitch AI (2016). Mechanisms of adaption to salinity stress in marine gastropods *Littorina saxatilis*: a proteomic analysis. Cell Tissue Biol.

[30] Wei L, Wang Q, Wu H, Ji C, Zhao J (2015). Proteomic and metabolomic responses of Pacific oyster *Crassostrea gigas* to elevated pCO2 exposure. J Proteome.

[31] Finn RD, Coggill P, Eberhardt RY, Eddy SR, Mistry J, Mitchell AL, Potter SC, Punta M, Qureshi M, Sangrador-Vegas A (2016). The Pfam protein families database: towards a more sustainable future. Nucleic Acids Res.

[32] The UniProt Consortium (2017). UniProt: the universal protein knowledgebase. Nucleic Acids Res.

[33] Sibbald SJ, Archibald JM (2017). More protist genomes needed. Nat Ecol Evol.

[34] Ortí G, Meyer A (1996). Molecular evolution of ependymin and the phylogenetic resolution of early divergences among euteleost fishes. Mol Biol Evol.

[35] Yang J, Yan R, Roy A, Xu D, Poisson J, Zhang Y (2014). The I-TASSER suite: protein structure and function prediction. Nat Methods.

[36] Pettersen EF, Goddard TD, Huang CC, Couch GS, Greenblatt DM, Meng EC, Ferrin TE (2004). UCSF chimera—a visualization system for exploratory research and analysis. J Comp Chem.

[37] Müller-Schmid A, Rinder H, Lottspeich F, Gertzen EM, Hoffmann W (1992). Ependymins from the cerebrospinal fluid of salmonid fish: gene structure and molecular characterization. Gene.

[38] Burki F, Kaplan M, Tikhonenkov DV, Zlatogursky V, Minh BQ, Radaykina LV, Smirnov A, Mylnikov AP, Keeling PJ (2016). Untangling the early diversification of eukaryotes: a phylogenomic study of the evolutionary origins of Centrohelida, Haptophyta and Cryptista. Proc R Soc B.

[39] Thormodsson FR, Antonian E, Grafstein B (1992). Extracellular proteins of goldfish optic tectum labeled by intraocular injection of 3H-proline. Exp Neurol.

[40] Schwarz H, Müller-Schmid A, Hoffman W (1993). Ultrastructural localization of ependymins in the endomeninx of the brain of the rainbow trout: possible association with collagen fibrils of the extracellular matrix. Cell Tissue Res.

[41] Hoffmann W (1994). Ependymins and their potential role in neuroplasticity and regeneration: calcium-binding meningeal glycoproteins of the cerebrospinal fluid and extracellular matrix. Int J BioChemiPhysics.

[42] Adams DS, Hasson B, Boyer-Boiteau A, El-Khishin A, Shashoua VE (2003). A peptide fragment of ependymin neurotrophic factor uses protein kinase C and the mitogen-activated protein kinase pathway to activate c-Jun N-terminal kinase and a functional AP-1 containing c-Jun and c-Fos proteins in mouse NB2a cells. J Neurosci Res.

[43] Saif S (2004). AP-1 is required for CMX-8933-induced SOD upregulation and is translocated in response to a human EPN mimetic. *PhD Thesis*.

[44] Schmidt R, Shashoua VE (1981). A radioimmunoassay for ependymins β and γ: two goldfish brain proteins involved in behavioral plasticity. J Neurochem.

[45] Thormodsson FR, Parker TS, Grafstein B (1992). Immunochemical studies of extracellular glycoproteins (X-GPs) of goldfish brain. Exp Neurol.

[46] Shashoua VE, Hesse GW, Milinazzo B (1990). Evidence for the in vivo polymerization of ependymin: a brain extracellular glycoprotein. Brain Res.

[47] Ganss B, Hoffmann W (2009). Calcium-induced conformational transition of trout ependymins monitored by tryptophan fluorescence. Open Biochem J.

[48] Haas BJ, Papanicolaou A, Yassour M, Grabherr M, Blood PD, Bowden J, Couger MB, Eccles D, Li B, Lieber M (2013). *De novo* transcript sequence reconstruction from RNA-seq using the trinity platform for reference generation and analysis. Nat Protoc.

[49] Eddy SR (1998). Profile hidden Markov models. Bioinformatics.

[50] Papadopoulos JS, Agarwala R (2007). COBALT: constraint-based alignment tool for multiple protein sequences. Bioinformatics.

[51] Larsson A (2014). AliView: a fast and lightweight alignment viewer and editor for large data sets. Bioinformatics.

[52] Stamatakis A (2006). RAxML-VI-HPC: maximum likelihood-based phylogenetic analyses with thousands of taxa and mixed models. Bioinformatics.

[53] Ronquist F, Teslenko M, van der Mark P, Ayres DL, Darling A, Höhna S, Larget B, Liu L, Suchard MA, Huelsenbeck JP (2012). MrBayes 3.2: efficient Bayesian phylogenetic inference and model choice across a large model space. Syst Biol.

[54] Huelsenbeck JP, Ronquist F (2001). MRBAYES: Bayesian inference of phylogenetic trees. Bioinformatics.

[55] Petersen TN, Brunak S, von Heijne G, Nielsen H (2011). SignalP 4.0: discriminating signal peptides from transmembrane regions. Nat Methods.

[56] Gupta R, Jung E, Brunak S. Prediction of N-glycosylation sites in human proteins. In preparation; 2004. http://www.cbs.dtu.dk/services/NetNGlyc/. Accessed 24 May 2018.

[57] Crooks GE, Hon G, Chandonia J-M, Brenner SE (2004). WebLogo: a sequence logo generator. Genome Res.

[58] R Core Team (2014). R: A Language and Environment for Statistical Computing.

[59] Wickham H (2009). ggplot2: elegant graphics for data analysis.

[60] Nordberg H, Cantor M, Dusheyko S, Hua S, Poliakov A, Shabalov I, Smirnova T, Grigoriev IV, Dubchak I (2014). The genome portal of the Department of Energy Joint Genome Institute: 2014 updates. Nucleic Acids Res.

[61] Huang S, Chen Z, Yan X, Yu T, Huang G, Yan Q, Pontarotti PA, Zhao H, Li J, Yang P (2014). Decelerated genome evolution in modern vertebrates revealed by analysis of multiple lancelet genomes. Nat Commun.

[62] Srivastava M, Simakov O, Chapman J, Fahey B, Gauthier MEA, Mitros T, Richards GS, Conaco C, Dacre M, Hellsten U (2010). The *Amphimedon queenslandica* genome and the evolution of animal complexity. Nature.

[63] Fernandez-Valverde SL, Calcino AD, Degnan BM (2015). Deep developmental transcriptome sequencing uncovers numerous new genes and enhances gene annotation in the sponge *Amphimedon queenslandica*. BMC Genomics.

[64] Horton T, Kroh A, Ahyong S, Bailly N, Boury-Esnault N, Brandão SN, Costello MJ, Gofas S, Hernandez F, Mees J, et al. World register of marine species (WoRMS): WoRMS Editorial Board; 2018. http://www.marinespecies.org. Accessed 24 May 2018.

[65] Robert V, Vu D, Amor ABH, van de Wiele N, Brouwer C, Jabas B, Szoke S, Dridi A, Triki M, Ben Daoud S (2013). MycoBank gearing up for new horizons. IMA Fungus.

[66] Giribet G, Edgecombe GD (2017). Current understanding of ecdysozoa and its internal phylogenetic relationships. Int Comp Biol.

[67] Luo Y-J, Kanda M, Koyanagi R, Hisata K, Akiyama T, Sakamoto H, Sakamoto T, Satoh N (2018). Nemertean and phoronid genomes reveal lophotrochozoan evolution and the origin of bilaterian heads. Nat Ecol Evol.

[68] Maddison DR, Schultz KS (2007). The tree of life web project.

[69] Whelan NV, Kocot KM, Moroz TP, Mukherjee K, Williams P, Paulay G, Moroz LL, Halanych KM (2017). Ctenophore relationships and their placement as the sister group to all other animals. Nat Ecol Evol.

[70] Simion P, Philippe H, Baurain D, Jager M, Richter DJ, Di Franco A, Roure B, Satoh N, Quéinnec E, Ereskovsky A (2017). A large and consistent phylogenomic dataset supports sponges as the sister group to all other animals. Curr Biol.

