# Supplementary material for: The evolution of ependymin-related proteins
Source: BMC Evol Biol. 2018 Dec 4;18:182. doi: 10.1186/s12862-018-1306-y (PMC6280359; doi:10.1186/s12862-018-1306-y)
Supplement: Supplementary file 2 — Note on incorrectly predicted gene models. Description of Data: Description of incorrectly predicted gene models within several metazoan genomes. (PDF 684 kb) [file 12862_2018_1306_MOESM2_ESM.pdf]

Additional file 2: Note on incorrectly predicted gene models.

In the course of this study it was noted that EPDR gene models within whole genome databases are frequently incorrect (Fig. S1).

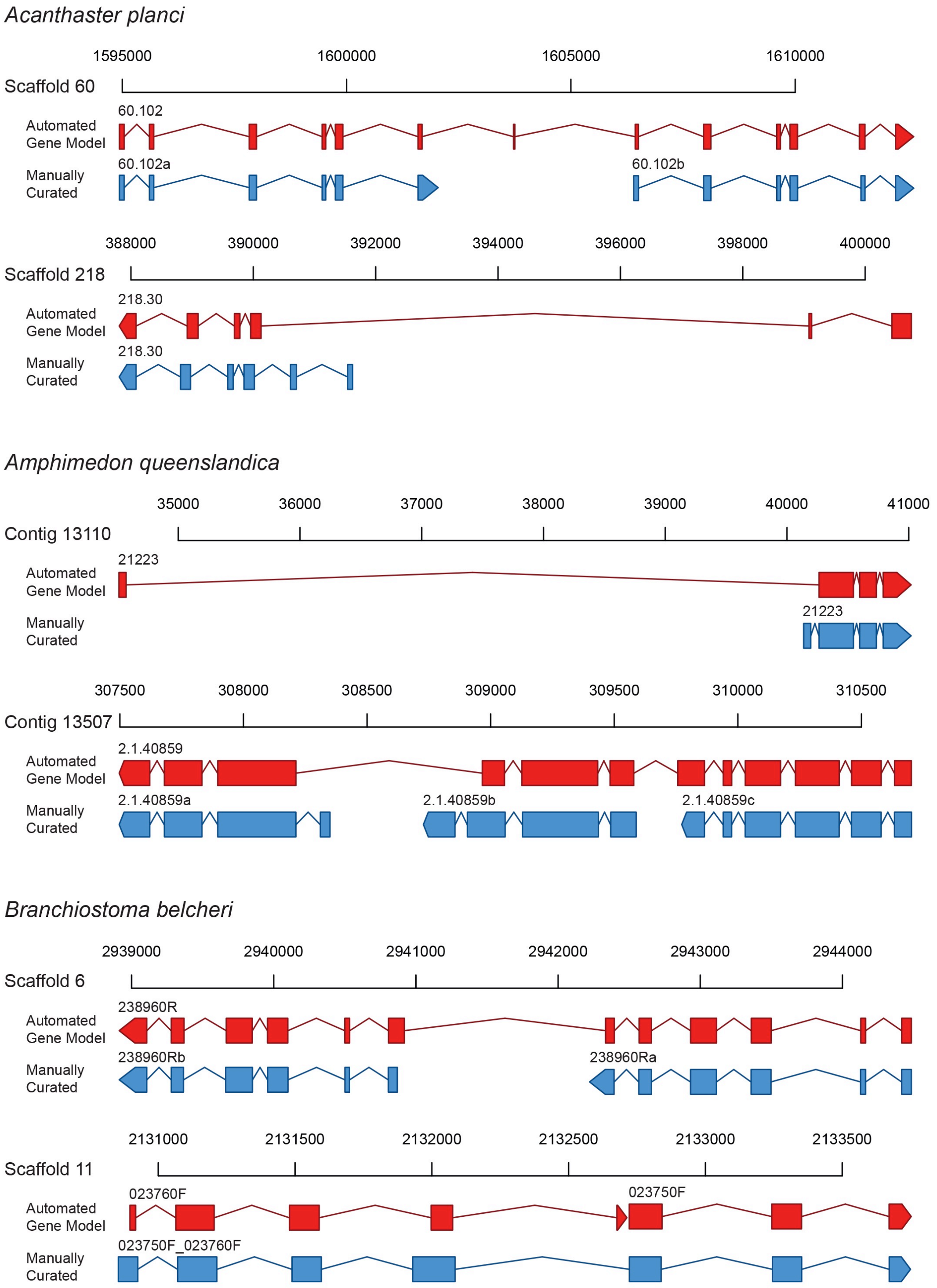

Fig. S1. Incorrectly predicted EPDR gene models in three different genomes. Automated gene model predictions are depicted in red, and manually curated models are presented in blue.

Observed errors include 1) multiple genes within one gene model, 2) single gene split into multiple gene models, 3) incorrect exon boundaries (often leading to missed stop codons), 4) incorrect start sites, and 5) missed exons. Gene model prediction for each of the genomes investigated (*Acanthaster planci*, *Amphimedon queenslandica*, and *Branchiostoma belcheri*) utilised *ab initio* prediction, transcriptome-based evidence, and homology modelling [1-3]. Given that there is good transcriptome evidence for the manually-curated gene models, at least for *A. planci* [1], it is unclear why the automated gene prediction performed so poorly (21 EPDRs found within automated gene models; 12 of these found to be incorrect upon manual curation).

1. Hall MR, Kocot KM, Baughman KW, Fernandez-Valverde SL, Gauthier MEA, Hatleberg WL, et al. The crown-of-thorns starfish genome as a guide for biocontrol of this coral reef pest. Nature. 2017;544:231-4. doi: 10.1038/nature22033.
2. Fernandez-Valverde SL, Calcino AD, Degnan BM. Deep developmental transcriptome sequencing uncovers numerous new genes and enhances gene annotation in the sponge *Amphimedon queenslandica*. BMC Genomics. 2015;16:387. doi: 10.1186/s12864-015-1588-z. PubMed PMID: 25975661.
3. Leiming, Y. *et al.* LanceletDB: an integrated genome database for lancelet, comparing domain types and combination in orthologues among lancelet and other species. Unpublished. Accessed 02/04/2018 http://genome.bucm.edu.cn/lancelet/.
