# Supplementary material for: The evolution of ependymin-related proteins
Source: BMC Evol Biol. 2018 Dec 4;18:182. doi: 10.1186/s12862-018-1306-y (PMC6280359; doi:10.1186/s12862-018-1306-y)
Supplement: Supplementary file 5 — Distribution of EPDR protein length. Description of Data: Violin plot displaying the range of sequence lengths exhibited by EPDR proteins. (PDF 837 kb) [file 12862_2018_1306_MOESM5_ESM.pdf]

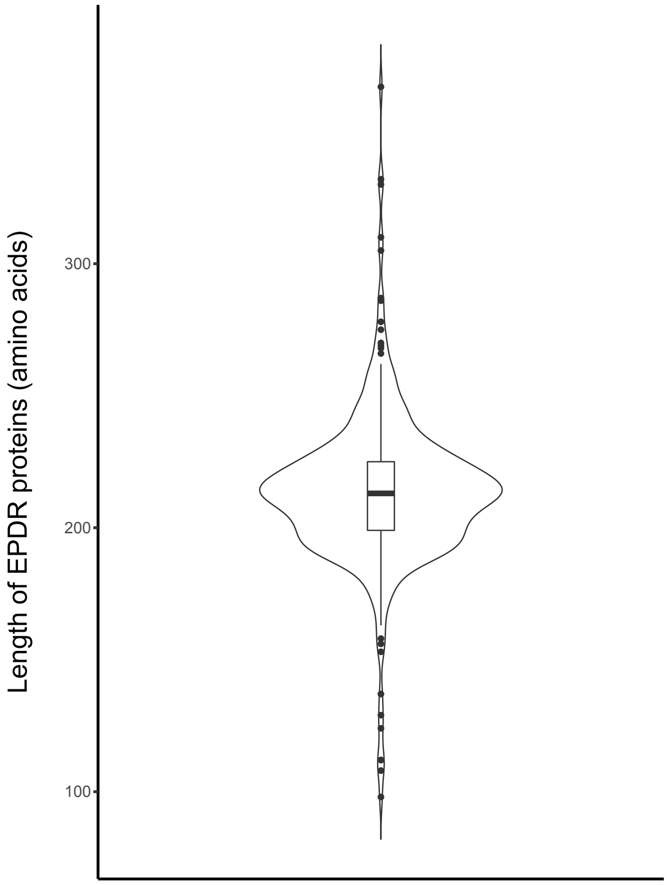

Additional file 5. Length of EPDR sequences identified in this study. The violin plot displays the length distribution of probable full-length (methionine start, signal peptide, and stop codon) EPDRs. The box plot in the centre indicates the median length (horizontal line, equal to 213 amino acids), as well as the second and third quartiles.
