# Supplementary material for: The evolution of ependymin-related proteins
Source: BMC Evol Biol. 2018 Dec 4;18:182. doi: 10.1186/s12862-018-1306-y (PMC6280359; doi:10.1186/s12862-018-1306-y)
Supplement: Supplementary file 6 — Results of phylogenetic analysis. Description of Data: Cladogram of the phylogenetic tree presented in Fig. 3, including bootstrap and posterior probability values. (PDF 1583 kb) [file 12862_2018_1306_MOESM6_ESM.pdf]

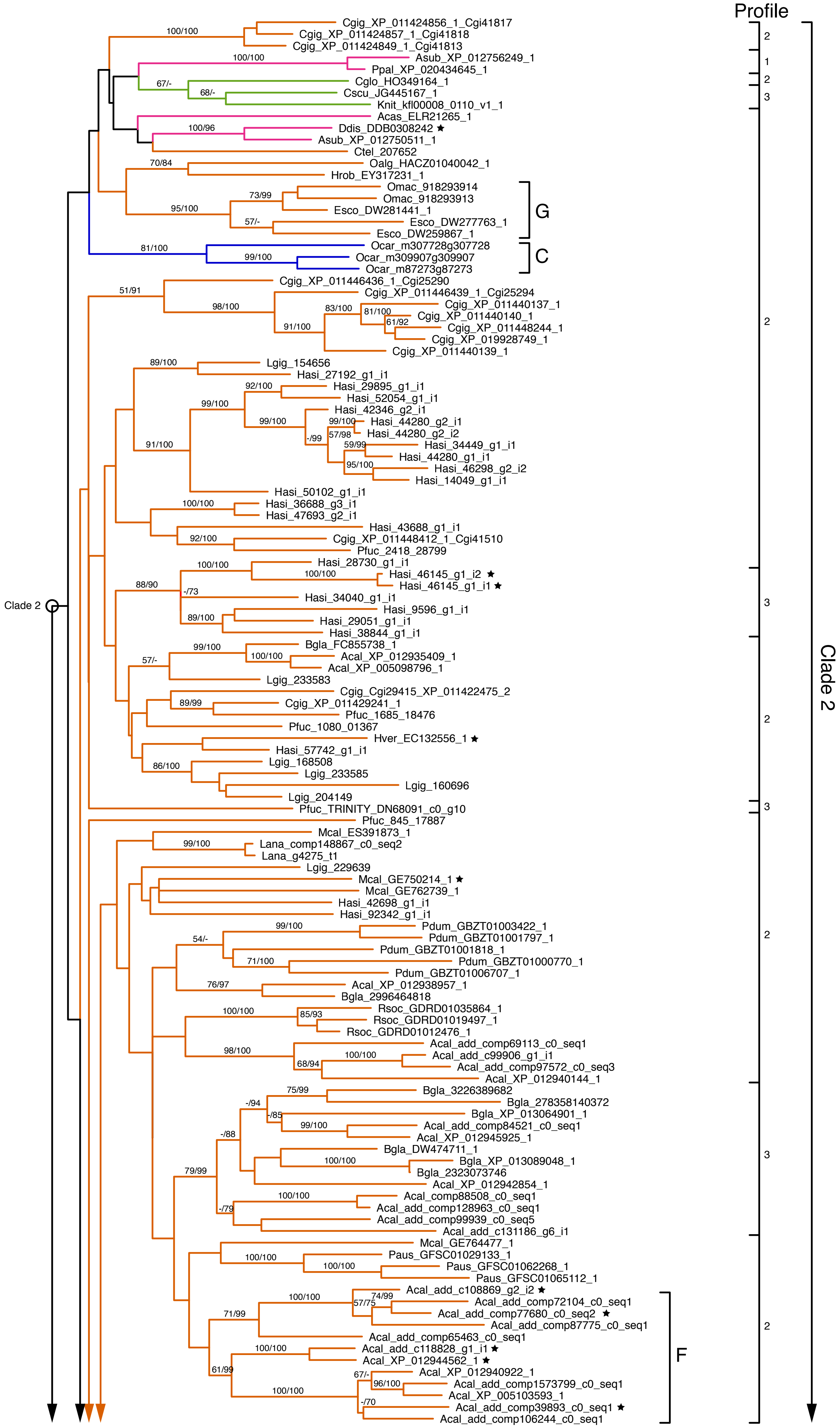
Profile
Clade 2
Cgig_XP_011424856_1_Cgi41817
Cgig_XP_011424857_1_Cgi41818
Cgig_XP_011424849_1_Cgi41813
Asub_XP_012756249_1
Ppal_XP_020434645_1
Cglo_HO349164_1
Cscu_JG445167_1
Knit_kfl00008_0110_v1_1
Acas_ELR21265_1
Ddis_DDB0308242 ★
Asub_XP_012750511_1
Ctel_207652
Oalg_HACZ01040042_1
Hrob_EY317231_1
Omac_918293914
Omac_918293913
Esco_DW281441_1
Esco_DW277763_1
Esco_DW259867_1
G
Ocar_m307728g307728
Ocar_m309907g309907
Ocar_m87273g87273
C
Cgig_XP_011446436_1_Cgi25290
Cgig_XP_011446439_1_Cgi25294
Cgig_XP_011440137_1
Cgig_XP_011440140_1
Cgig_XP_011448244_1
Cgig_XP_019928749_1
Cgig_XP_011440139_1
Lgig_154656
Hasi_27192_g1_i1
Hasi_29895_g1_i1
Hasi_52054_g1_i1
Hasi_42346_g2_i1
Hasi_44280_g2_i1
Hasi_44280_g2_i2
Hasi_34449_g1_i1
Hasi_44280_g1_i1
Hasi_46298_g2_i2
Hasi_14049_g1_i1
Hasi_50102_g1_i1
Hasi_36688_g3_i1
Hasi_47693_g2_i1
Hasi_43688_g1_i1
Cgig_XP_011448412_1_Cgi41510
Pfuc_2418_28799
Hasi_28730_g1_i1
Hasi_46145_g1_i2 ★
Hasi_46145_g1_i1 ★
Hasi_34040_g1_i1
Hasi_9596_g1_i1
Hasi_29051_g1_i1
Hasi_38844_g1_i1
Bgla_FC855738_1
Acal_XP_012935409_1
Acal_XP_005098796_1
Lgig_233583
Cgig_Cgi29415_XP_011422475_2
Cgig_XP_011429241_1
Pfuc_1685_18476
Pfuc_1080_01367
Hver_EC132556_1 ★
Hasi_57742_g1_i1
Lgig_168508
Lgig_233585
Lgig_160696
Lgig_204149
Pfuc_TRINITY_DN68091_c0_g10
Pfuc_845_17887
Mcal_ES391873_1
Lana_comp148867_c0_seq2
Lana_g4275_t1
Lgig_229639
Mcal_GE750214_1 ★
Mcal_GE762739_1
Hasi_42698_g1_i1
Hasi_92342_g1_i1
Pdum_GBZT01003422_1
Pdum_GBZT01001797_1
Pdum_GBZT01001818_1
Pdum_GBZT01000770_1
Pdum_GBZT01006707_1
Acal_XP_012938957_1
Bgla_2996464818
Rsoc_GDRD01035864_1
Rsoc_GDRD01019497_1
Rsoc_GDRD01012476_1
Acal_add_comp69113_c0_seq1
Acal_add_c99906_g1_i1
Acal_add_comp97572_c0_seq3
Acal_XP_012940144_1
Bgla_3226389682
Bgla_278358140372
Bgla_XP_013064901_1
Acal_add_comp84521_c0_seq1
Acal_XP_012945925_1
Bgla_DW474711_1
Bgla_XP_013089048_1
Bgla_2323073746
Acal_XP_012942854_1
Acal_comp88508_c0_seq1
Acal_add_comp128963_c0_seq1
Acal_add_comp99939_c0_seq5
Acal_add_c131186_g6_i1
Mcal_GE764477_1
Paus_GFSC01029133_1
Paus_GFSC01062268_1
Paus_GFSC01065112_1
Acal_add_c108869_g2_i2 ★
Acal_add_comp72104_c0_seq1
Acal_add_comp77680_c0_seq2 ★
Acal_add_comp87775_c0_seq1
Acal_add_comp65463_c0_seq1
Acal_add_c118828_g1_i1 ★
Acal_XP_012944562_1 ★
Acal_XP_012940922_1
Acal_add_comp1573799_c0_seq1
Acal_XP_005103593_1
Acal_add_comp39893_c0_seq1 ★
Acal_add_comp106244_c0_seq1
F
Clade 2
2
1
2
3
2
3
2
3
2
3
2

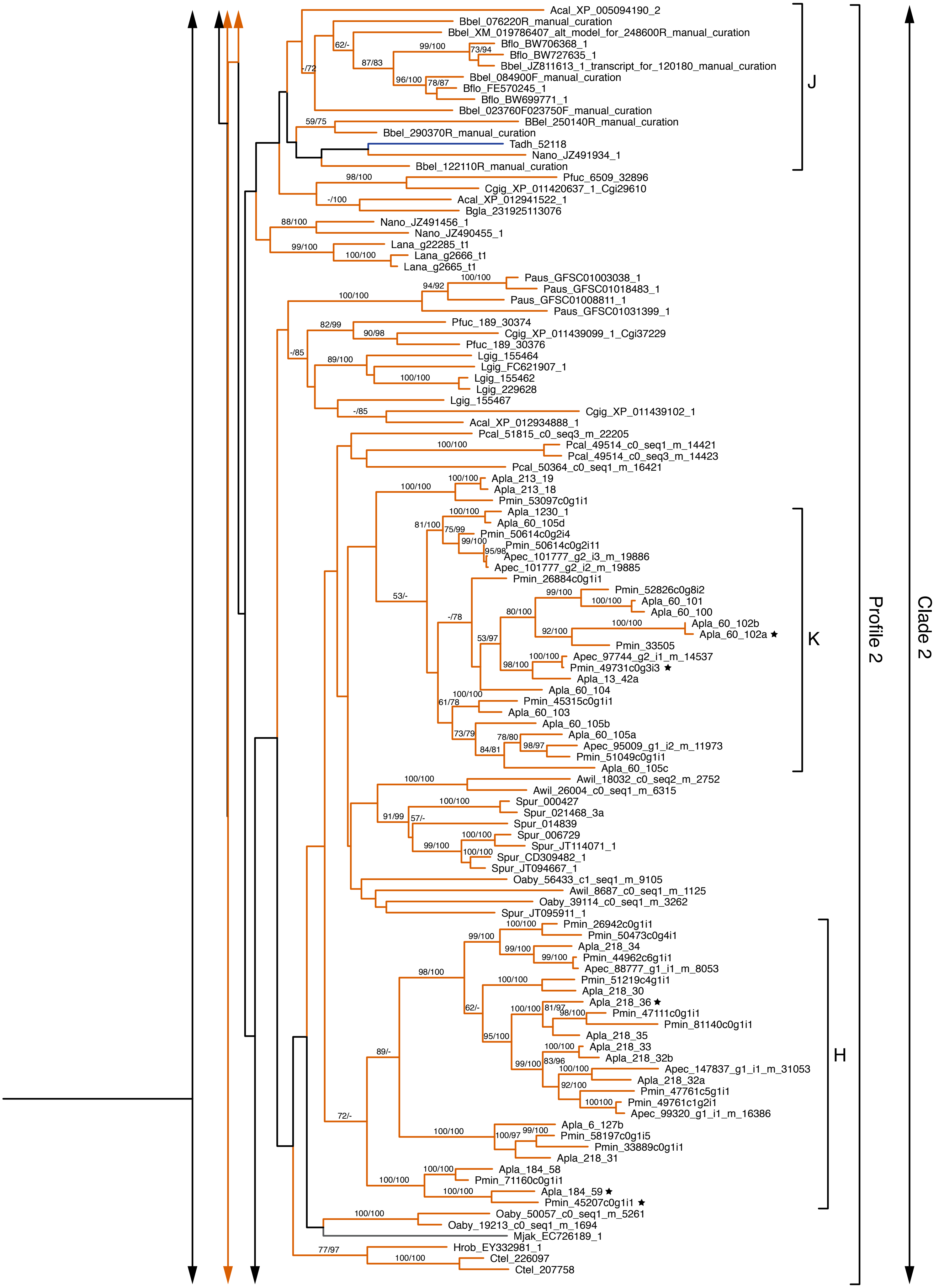
Acal_XP_005094190_2
Bbel_076220R_manual_curation
Bbel_XM_019786407_alt_model_for_248600R_manual_curation
Bflo_BW706368_1
Bflo_BW727635_1
Bbel_JZ811613_1_transcript_for_120180_manual_curation
Bbel_084900F_manual_curation
Bflo_FE570245_1
Bflo_BW699771_1
Bbel_023760F023750F_manual_curation
BBel_250140R_manual_curation
Bbel_290370R_manual_curation
Tadh_52118
Nano_JZ491934_1
Bbel_122110R_manual_curation
J
Pfuc_6509_32896
Cgig_XP_011420637_1_Cgi29610
Acal_XP_012941522_1
Bgla_231925113076
Nano_JZ491456_1
Nano_JZ490455_1
Lana_g22285_t1
Lana_g2666_t1
Lana_g2665_t1
Paus_GFSC01003038_1
Paus_GFSC01018483_1
Paus_GFSC01008811_1
Paus_GFSC01031399_1
Pfuc_189_30374
Cgig_XP_011439099_1_Cgi37229
Pfuc_189_30376
Lgig_155464
Lgig_FC621907_1
Lgig_155462
Lgig_229628
Lgig_155467
Cgig_XP_011439102_1
Acal_XP_012934888_1
Pcal_51815_c0_seq3_m_22205
Pcal_49514_c0_seq1_m_14421
Pcal_49514_c0_seq3_m_14423
Pcal_50364_c0_seq1_m_16421
Apla_213_19
Apla_213_18
Pmin_53097c0g1i1
Apla_1230_1
Apla_60_105d
Pmin_50614c0g2i4
Pmin_50614c0g2i11
Apec_101777_g2_i3_m_19886
Apec_101777_g2_i2_m_19885
Pmin_26884c0g1i1
Pmin_52826c0g8i2
Apla_60_101
Apla_60_100
Apla_60_102b
Apla_60_102a ★
Pmin_33505
Apec_97744_g2_i1_m_14537
Pmin_49731c0g3i3 ★
Apla_13_42a
Apla_60_104
Pmin_45315c0g1i1
Apla_60_103
Apla_60_105b
Apla_60_105a
Apec_95009_g1_i2_m_11973
Pmin_51049c0g1i1
Apla_60_105c
K
Awil_18032_c0_seq2_m_2752
Awil_26004_c0_seq1_m_6315
Spur_000427
Spur_021468_3a
Spur_014839
Spur_006729
Spur_JT114071_1
Spur_CD309482_1
Spur_JT094667_1
Oaby_56433_c1_seq1_m_9105
Awil_8687_c0_seq1_m_1125
Oaby_39114_c0_seq1_m_3262
Spur_JT095911_1
Pmin_26942c0g1i1
Pmin_50473c0g4i1
Apla_218_34
Pmin_44962c6g1i1
Apec_88777_g1_i1_m_8053
Pmin_51219c4g1i1
Apla_218_30
Apla_218_36 ★
Pmin_47111c0g1i1
Pmin_81140c0g1i1
Apla_218_35
Apla_218_33
Apla_218_32b
Apec_147837_g1_i1_m_31053
Apla_218_32a
Pmin_47761c5g1i1
Pmin_49761c1g2i1
Apec_99320_g1_i1_m_16386
Apla_6_127b
Pmin_58197c0g1i5
Pmin_33889c0g1i1
Apla_218_31
Apla_184_58
Pmin_71160c0g1i1
Apla_184_59 ★
Pmin_45207c0g1i1 ★
H
Oaby_50057_c0_seq1_m_5261
Oaby_19213_c0_seq1_m_1694
Mjak_EC726189_1
Hrob_EY332981_1
Ctel_226097
Ctel_207758
Profile 2
Clade 2

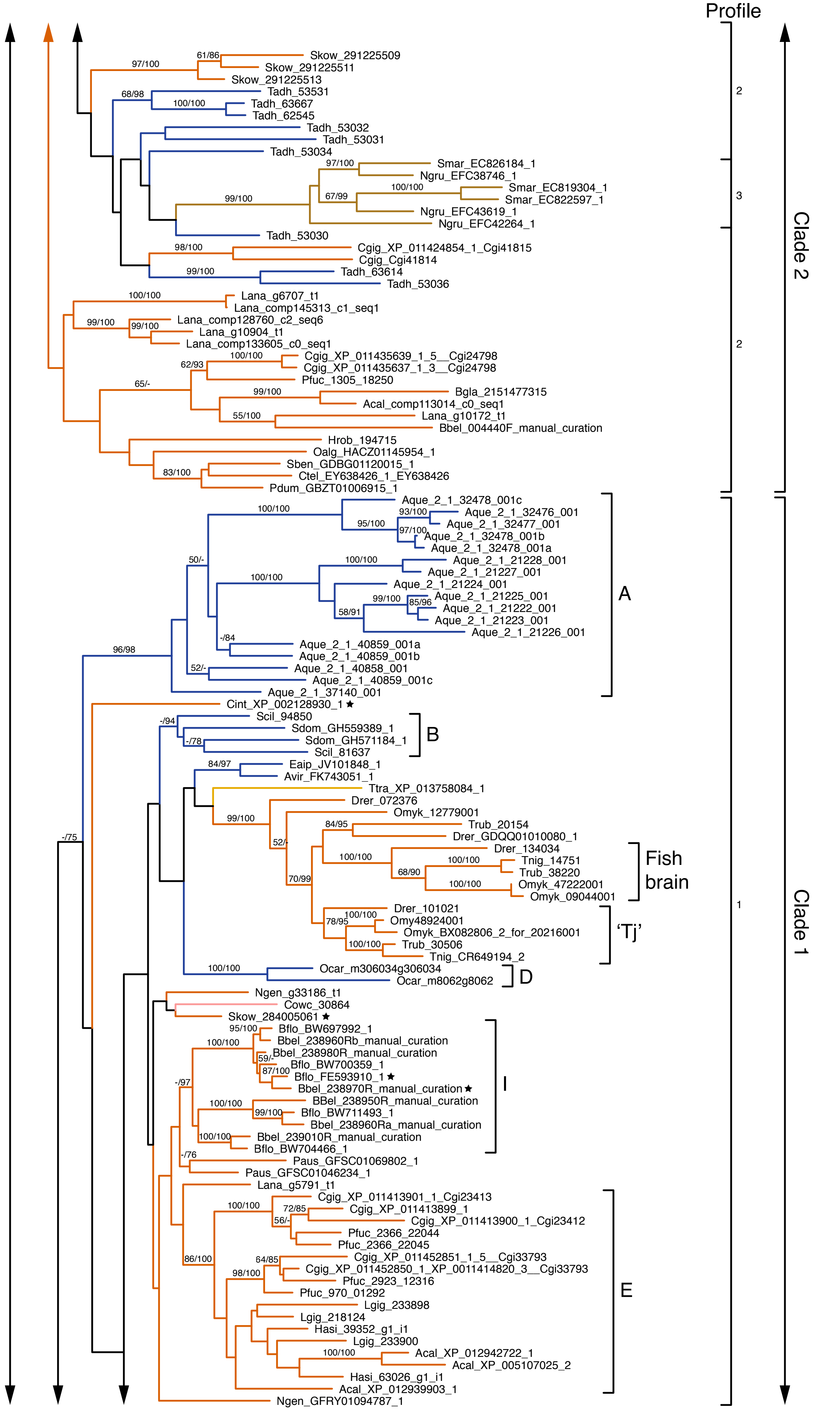

Profile
Skow_291225509
Skow_291225511
Skow_291225513
Tadh_53531
Tadh_63667
Tadh_62545
Tadh_53032
Tadh_53031
Tadh_53034
Smar_EC826184_1
Ngru_EFC38746_1
Smar_EC819304_1
Smar_EC822597_1
Ngru_EFC43619_1
Ngru_EFC42264_1
Tadh_53030
Cgig_XP_011424854_1_Cgi41815
Cgig_Cgi41814
Tadh_63614
Tadh_53036
Lana_g6707_t1
Lana_comp145313_c1_seq1
Lana_comp128760_c2_seq6
Lana_g10904_t1
Lana_comp133605_c0_seq1
Cgig_XP_011435639_1_5__Cgi24798
Cgig_XP_011435637_1_3__Cgi24798
Pfuc_1305_18250
Bgla_2151477315
Acal_comp113014_c0_seq1
Lana_g10172_t1
Bbel_004440F_manual_curation
Hrob_194715
Oalg_HACZ01145954_1
Sben_GDBG01120015_1
Ctel_EY638426_1_EY638426
Pdum_GBZT01006915_1
Aque_2_1_32478_001c
Aque_2_1_32476_001
Aque_2_1_32477_001
Aque_2_1_32478_001b
Aque_2_1_32478_001a
Aque_2_1_21228_001
Aque_2_1_21227_001
Aque_2_1_21224_001
Aque_2_1_21225_001
Aque_2_1_21222_001
Aque_2_1_21223_001
Aque_2_1_21226_001
Aque_2_1_40859_001a
Aque_2_1_40859_001b
Aque_2_1_40858_001
Aque_2_1_40859_001c
Aque_2_1_37140_001
Cint_XP_002128930_1 ★
Scil_94850
Sdom_GH559389_1
Sdom_GH571184_1
Scil_81637
Eaip_JV101848_1
Avir_FK743051_1
Ttra_XP_013758084_1
Drer_072376
Omyk_12779001
Trub_20154
Drer_GDQQ01010080_1
Drer_134034
Tnig_14751
Trub_38220
Omyk_47222001
Omyk_09044001
Drer_101021
Omy48924001
Omyk_BX082806_2_for_20216001
Trub_30506
Tnig_CR649194_2
Ocar_m306034g306034
Ocar_m8062g8062
Ngen_g33186_t1
Cowc_30864
Skow_284005061 ★
Bflo_BW697992_1
Bbel_238960Rb_manual_curation
Bbel_238980R_manual_curation
Bflo_BW700359_1
Bflo_FE593910_1 ★
Bbel_238970R_manual_curation ★
BBel_238950R_manual_curation
Bflo_BW711493_1
Bbel_238960Ra_manual_curation
Bbel_239010R_manual_curation
Bflo_BW704466_1
Paus_GFSC01069802_1
Paus_GFSC01046234_1
Lana_g5791_t1
Cgig_XP_011413901_1_Cgi23413
Cgig_XP_011413899_1
Cgig_XP_011413900_1_Cgi23412
Pfuc_2366_22044
Pfuc_2366_22045
Cgig_XP_011452851_1_5__Cgi33793
Cgig_XP_011452850_1_XP_0011414820_3__Cgi33793
Pfuc_2923_12316
Pfuc_970_01292
Lgig_233898
Lgig_218124
Hasi_39352_g1_i1
Lgig_233900
Acal_XP_012942722_1
Acal_XP_005107025_2
Hasi_63026_g1_i1
Acal_XP_012939903_1
Ngen_GFRY01094787_1
97/100
61/86
68/98
100/100
97/100
100/100
67/99
99/100
98/100
99/100
100/100
99/100
99/100
100/100
62/93
65/-
99/100
55/100
83/100
100/100
93/100
95/100
97/100
50/-
100/100
100/100
99/100
85/96
58/91
96/98
-/84
52/-
-/75
-/94
-/78
84/97
99/100
84/95
52/-
100/100
100/100
68/90
100/100
70/99
100/100
78/95
100/100
100/100
95/100
100/100
59/-
87/100
-/97
100/100
99/100
100/100
-/76
100/100
72/85
56/-
86/100
64/85
98/100
100/100
2
3
2
1
A
B
Fish brain
'Tj'
D
I
E
Clade 2
Clade 1

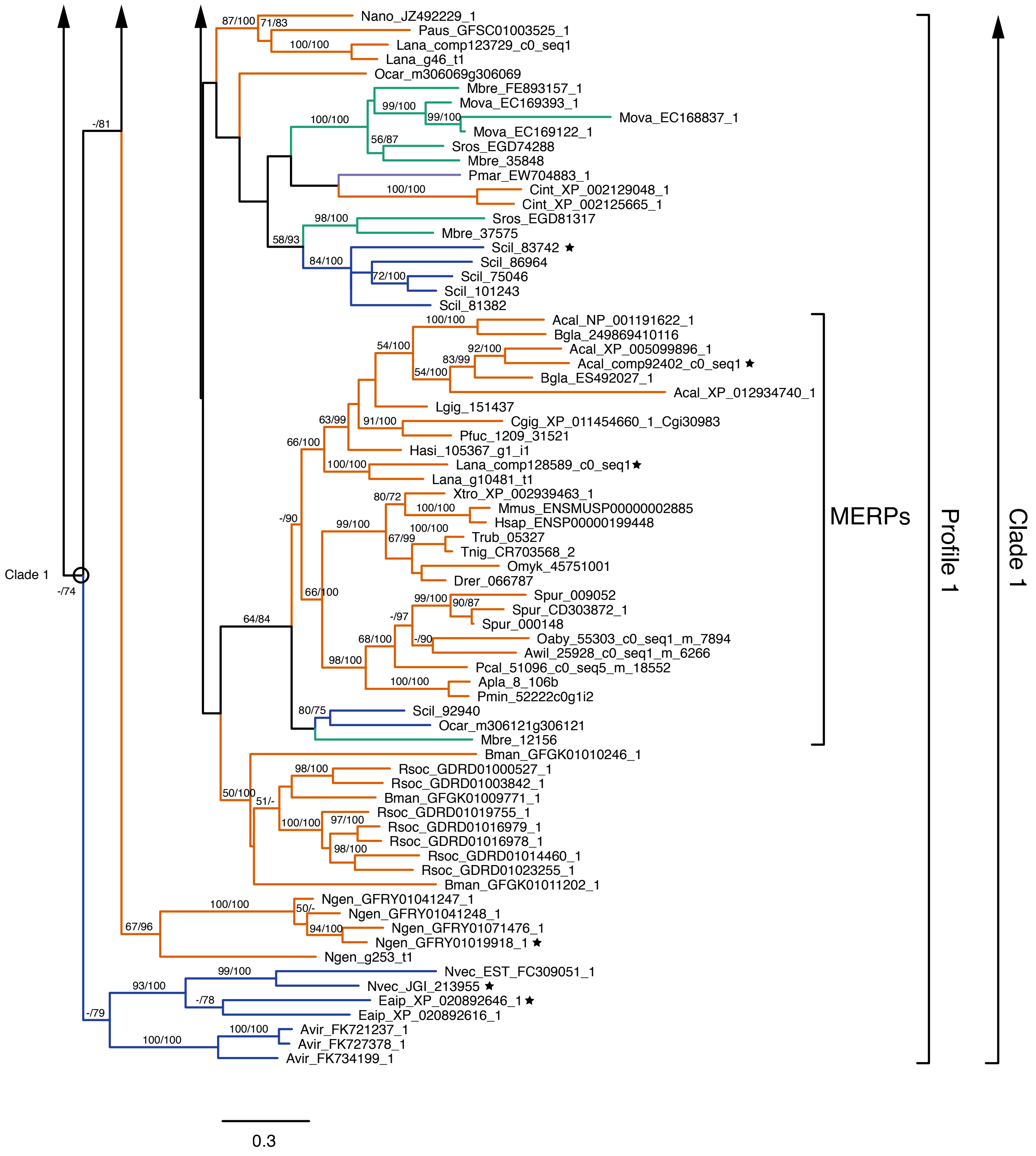

Additional file 6. Complete phylogenetic analysis corresponding to that presented in Fig. 3 (maximum likelihood). Colouring of the branches corresponds to the phylogenetic groups to which the species belong, and follows the scheme established in Figure 1. Values above branches indicate maximum likelihood bootstrap values (when over 50), followed by Bayesian posterior probabilities (when over 70). The scale bar reflects the number of substitutions per site. Full length sequences that do not contain predicted N-glycosylation sites are indicated by a star. Species abbreviations: Acal=*Aplysia californica*, Acas=*Acanthamoeba castellanii*, Apec=*Asterina pectinifera (Patiria)*, Apla=*Acanthaster planci*, Aque=*Amphimedon queenslandica*, Asub=*Acytostelium subglobosum*, Avir=*Anemonia viridis*, Awil=*Apometra wilsoni (Aporometra)*, Bbel=*Branchiostoma belcheri*, Bflo=*Branchiostoma floridae*, Bgla=*Biomphalaria glabrata*, Bman=*Brachionus manjavacas*, Cgig=*Crassostrea gigas* , Cglo=*Chaetosphaeridium globosum*, Cint=*Ciona intestinalis*, Cowc=*Capsaspora owczarzaki*, Cscu=*Coleochaete scutata*, Ctel=*Capitella teleta*, Ddis=*Dictyostelium*

discoideum, Drer=*Danio rerio*, Eaip=*Exaiptasia pallida*, Esco=*Euprymna scolopes*, Hasi=*Haliotis asinina*, Hrob=*Helobdella robusta*, Hsap=*Homo sapiens*, Hver=*Hartmannella vermiformis*, Knit=*Klebsormidium nitens (flaccidum)*, Lana=*Lingula anatina*, Lgig=*Lottia gigantea*, Mbre=*Monosiga brevicollis*, Mcal=*Mytilus californianus*, Mjak=*Malawimonas jakobiformis*, Mmus=*Mus musculus*, Mova=*Monosiga ovata*, Nano=*Novocrania anomala*, Ngen=*Notospermus geniculatus*, Ngru=*Naegleria gruberi*, Nvec=*Nematostella vectensis*, Oaby=*Ophiactis abyssicola*, Ocar=*Oscarella carmela*, Oalg=*Olavius algarvensis*, Omac=*Octopus maculoides (bimaculoides)*, Omyk=*Oncorhynchus mykiss*, Paus=*Phoronis australis*, Pcal=*Parastichopus californicus (Apostichopus)*, Pdum=*Platynereis dumerilii,* Pfuc=*Pinctada fucata*, Pmar=*Paracercomonas marina*, Pmin=*Patiria miniata*, Ppal=*Polysphondylium pallidum*, Rsoc=*Rotaria socialis*, Sben=*Streblospio benedicti*, Scil=*Sycon ciliatum*, Sdom=*Suberites domuncula*, Skow=*Saccoglossus kowalevskii*, Smar=*Sawyeria marylandensis*, Spur=*Strongylocentrotus purpuratus*, Sros=*Salpingoeca rosetta*, Tadh=*Trichoplax adhaerens*, Tnig=*Tetraodon nigroviridis*, Trub=*Takifugu rubripes*, Ttra=*Thecamonas trahens*, Xtro=*Xenopus tropicalis*.
