# Supplementary material for: The evolution of ependymin-related proteins
Source: BMC Evol Biol. 2018 Dec 4;18:182. doi: 10.1186/s12862-018-1306-y (PMC6280359; doi:10.1186/s12862-018-1306-y)
Supplement: Supplementary file 8 — Clustering of EPDR genes in genomes. Description of Data: Schematic indicating clustering of EPDR genes on scaffolds in the Acanthaster planci, Amphimedon queenslandica and Branchiostoma belcheri genomes. (PDF 912 kb) [file 12862_2018_1306_MOESM8_ESM.pdf]

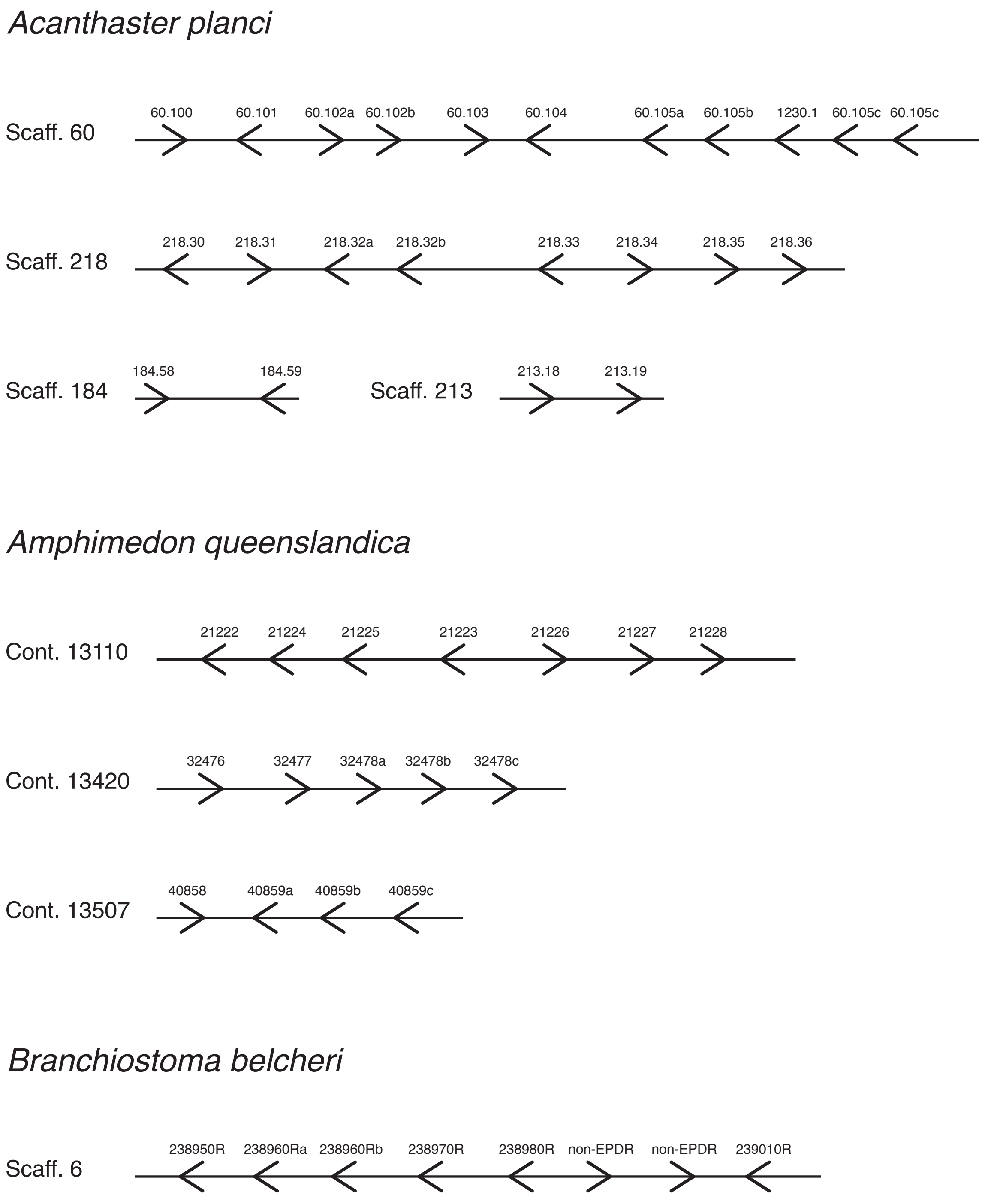

Additional File 8. Clustering of EPDR genes in genomes. Many EPDR genes are clustered on scaffolds within *A. planci, A. queenslandica* and *B. belcheri* genomes. Arrowheads indicate the direction of transcription. Not to scale.
