# Supplementary material for: The evolution of ependymin-related proteins
Source: BMC Evol Biol. 2018 Dec 4;18:182. doi: 10.1186/s12862-018-1306-y (PMC6280359; doi:10.1186/s12862-018-1306-y)
Supplement: Supplementary file 7 — Results of phylogenetic analysis on reduced dataset. Description of Data: Summary tree of phylogenetic analysis conducted after the removal of problematic taxa. (PDF 424 kb) [file 12862_2018_1306_MOESM7_ESM.pdf]

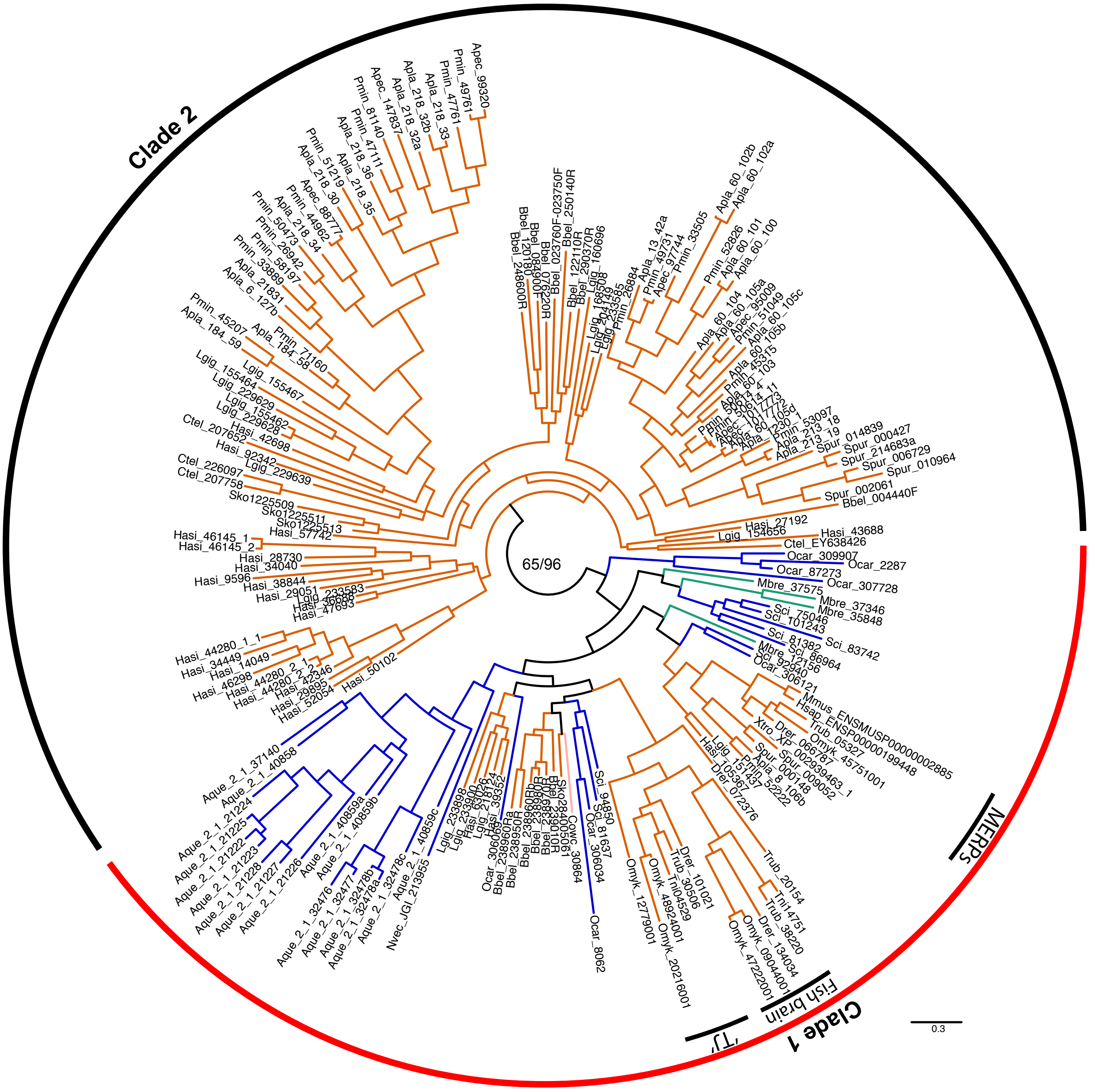

Additional file 7. EPDR phylogenetic analysis with reduced taxa (maximum likelihood). This analysis produces higher support for the presence of the two major EPDR clades. The values within the central circle of the tree indicate a maximum likelihood bootstrap value of 65 and a Bayesian posterior probablility of 96% for the division into two clades. Colouring of the branches corresponds to the phylogenetic groups to which the species belong, and follows the scheme established in Figure 1. The scale bar reflects the number of substitutions per site. Species abbreviations: Apec=*Asterina pectinifera*, Apla=*Acanthaster planci*, Aque=*Amphimedon queenslandica*, Bbel=*Branchiostoma belcheri*, Cowc=*Capsaspora owczarzaki*, Ctel=*Capitella teleta*, Drer=*Danio rerio*, Hasi=*Haliotis asinina*, Hsap=*Homo sapiens*, Lgig=*Lottia gigantea*, Mbre=*Monosiga brevicollis*, Mmus=*Mus musculus*, Nvec=*Nematostella vectensis*, Ocar= *Oscarella carmela*, Omyk=*Oncorhynchus mykiss*, Pmin=*Patiria miniata*, Scil=*Sycon ciliatum*, Skow=*Saccoglossus kowalevskii*, Spur=*Strongylocentrotus purpuratus*,Tnig=*Tetraodon nigroviridis*, Trub=*Takifugu rubripes*, Xtro=*Xenopus tropicalis*.
