# Supplementary material for: The evolution of ependymin-related proteins
Source: BMC Evol Biol. 2018 Dec 4;18:182. doi: 10.1186/s12862-018-1306-y (PMC6280359; doi:10.1186/s12862-018-1306-y)
Supplement: Supplementary file 9 — Predicted model scores and ligands for representative EPDR sequences. Description of Data: Table showing clade membership, model C-Scores, and predicted ligands of selected EPDR sequences. (PDF 60 kb) [file 12862_2018_1306_MOESM9_ESM.pdf]

Additional file 9. Predicted model scores and ligands for representative EPDR sequences.

Protein	Clade/ profile	C-Score (model)	Predicted ligands	C-Score (ligand)
D. rerio 134034	1/1	-1.20	Phenylpyruvic acid	0.07
			Tetraethylene glycol monooctyl ether	0.05
M. musculus ENSMUSP00000002885	1/1	-0.78	Tetraethylene glycol	0.37
			Peptide	0.06
			Phenylpyruvic acid	0.06
			Tetraethylene glycol monooctyl ether	0.04
C. owczarzaki 30864	1/1	-0.64	Tetraethylene glycol	0.21
			Tetraethylene glycol monooctyl ether	0.07
S. kowalevskii 91225509	2/2	-2.85	Tetraethylene glycol monooctyl ether	0.10
			Phenylpyruvic acid	0.10
C. globosum HO349164	2/2	-3.03	Tetraethylene glycol	0.23
			Tetraethylene glycol monooctyl ether	0.07
			Phenylpyruvic acid	0.06
N. gruberi EFC42264	2/3	-2.44	Tetraethylene glycol	0.15
			Tetraethylene glycol monooctyl ether	0.15
A. californica c13118	2/3	-1.74	Tetraethylene glycol	0.13
			Tetraethylene glycol monooctyl ether	0.07

Details of 3D modelling of EPDR sequences in I-TASSER. C-score (model) is the confidence score of the model on a scale of -5 to 2, where higher values signify higher confidence. C-score (ligand) denotes the confidence of the ligand prediction on a scale of 0 to 1, with higher values again signifying higher confidence.
